# Melatonin-encapsuled silk fibroin electrospun nanofibers promote vascularized bone regeneration through regulation of osteogenesis-angiogenesis coupling

**DOI:** 10.1016/j.mtbio.2024.100985

**Published:** 2024-02-02

**Authors:** Lei Deng, Mingzhuang Hou, Nanning Lv, Quan Zhou, Xi Hua, Xiayu Hu, Xiaoyang Ge, Xuesong Zhu, Yong Xu, Huilin Yang, Xi Chen, Hao Liu, Fan He

**Affiliations:** a Department of Orthopaedics, First Affiliated Hospital of Soochow University, Soochow University, Suzhou, 215006, China; b Orthopaedic Institute, Suzhou Medical College, Soochow University, Suzhou, 215000, China; c Department of Orthopedic Surgery, Lianyungang Clinical College of Xuzhou Medical University, Lianyungang, 222003, China; d Department of Pathology, Third Affiliated Hospital of Soochow University, Changzhou, 213003, China

**Keywords:** Periosteum, Electrospinning, Melatonin, Angiogenesis, Osteogenesis, Coupling

## Abstract

The repair of critical-sized bone defects poses a significant challenge due to the absence of periosteum, which plays a crucial role in coordinating the processes of osteogenesis and vascularization during bone healing. Herein, we hypothesized that melatonin-encapsuled silk Fibronin electrospun nanofibers (SF@MT) could provide intrinsic induction of both osteogenesis and angiogenesis, thereby promoting vascularized bone regeneration. The sustained release of melatonin from the SF@MT nanofibers resulted in favorable biocompatibility and superior osteogenic induction of bone marrow mesenchymal stem cells (BMMSCs). Interestingly, melatonin promoted the migration and tube formation of human umbilical vein endothelial cells (HUVECs) in a BMMSC-dependent manner, potentially through the upregulation of vascular endothelial growth factor (VEGFA) expression in SF@MT-cultured BMMSCs. SF@MT nanofibers enhanced the BMMSC-mediated angiogenesis by activating the PI3K/Akt signaling pathway. *In vivo* experiments indicated that the implantation of SF@MT nanofibers into rat critical-sized calvarial defects significantly enhances the production of bone matrix and the development of new blood vessels, leading to an accelerated process of vascularized bone regeneration. Consequently, the utilization of melatonin-encapsulated silk Fibronin electrospun nanofibers shows great promise as a potential solution for artificial periosteum, with the potential to regulate the coupling of osteogenesis and angiogenesis in critical-sized bone defect repair.

## Graphical abstract

**Figure:**
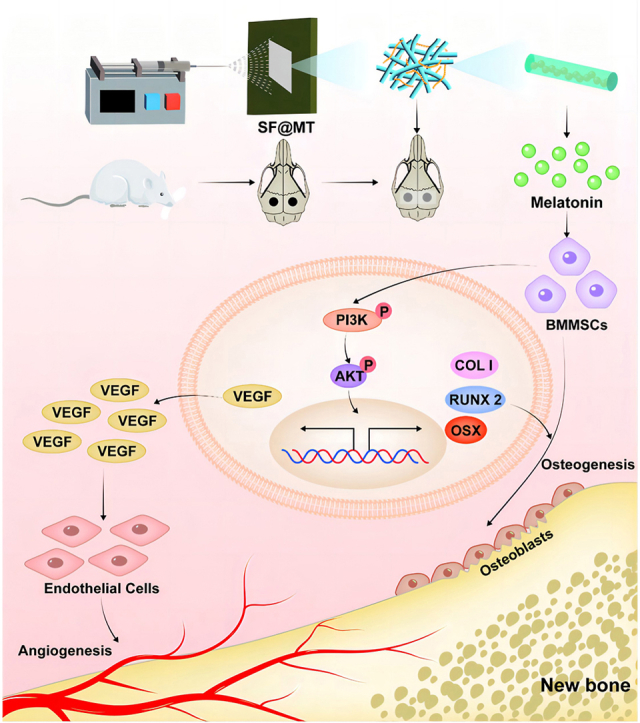

## 1 Introduction

The increasing prevalence of bone defects resulting from severe trauma, surgical resection, and radical tumor resection poses a significant obstacle in the field of clinical practice [1]. Currently, the main approaches utilized for addressing bone defects include autologous bone grafts, allograft bone grafts, and traction osteogenesis [2]. Nevertheless, the outcomes of these methods are deemed inadequate for clinical applications due to their inherent limitations. For instance, the acquisition of autologous bone necessitates an extra surgical intervention and is further hindered by limitations in quantity and substantial expenses [3]. Allograft bone grafting presents concerns regarding immunogenicity, rejection reactions, potential transmission of infections, and high costs [4]. As well, distraction osteogenesis has some limitations, such as complications associated with poor healing and re-fracture, the lengthy process required during treatment and consolidation, and the psychological and health effects on patients [5].

Bone defects are frequently associated with the lack of periosteum, a connective tissue membrane that encloses bone tissue and plays a vital role in bone tissue development and repair. Generally, periosteum consists of an inner and outer layer, primarily composed of collagen and elastic fibers, along with a comprehensive vascular network that offers structural support and blood circulation to both the periosteum and bone tissues [6]. Notably, the periosteum plays a crucial role in facilitating osteogenesis by supplying nutrients via an extensive network of capillaries. The activation of vascular endothelial cells coincides with the migration, proliferation, and differentiation of osteoblast progenitors at the site of repair, thereby restoring nutrient and oxygen supply to facilitate the repair of bone defects [7]. Recent studies have highlighted the fundamental aspects of vessel formation, including vasculogenic assembly, vessel sprouting, lumen formation and vascular remodeling [8,9]. The connection between angiogenesis and osteogenesis is evident during the healing of bone fractures, as a timely and coordinated angiogenic response is of vital importance for successful bone repair. Therefore, the development of artificial periosteum holds great potential as a strategy to expedite bone healing and enhance the quality of bone repair.

In recent times, electrospinning has gained prominence as a prevalent technique for producing fibrous and membrane-like networks spanning from micro-to nanometer dimensions. The electrospinning technique has garnered considerable interest within the field of bone tissue engineering due to its utilization of diverse biomaterials, including synthetic polymers, natural biomacromolecules, and composite materials [10]. Silk fibroin (SF), a naturally derived protein obtained from silk, has demonstrated advantageous mechanical characteristics and biocompatibility, rendering it a promising contender for bone tissue engineering applications [11]. A study has documented the development of a non-woven film through the integration of SF and polyhydroxyalkanoates via the electrospinning technique. Although SF nanofibers alone failed to significantly increased osteogenic-marker gene expression in the cultivation of human MSCs, the application of this composite electrospun film through the blending of polyhydroxyalkanoates and SF has proven to be beneficial in promoting osteogenic differentiation and matrix mineralization [12].

Melatonin, a neuroendocrine hormone synthesized by the pineal gland, has been shown to play a crucial role in regulating circadian rhythm, immunity, and the removal of free radicals [13]. Extensive research has demonstrated that melatonin serves as a significant regulator of bone metabolism [14], exerting its effects through the facilitation of osteoblast-mediated bone matrix formation and mineralization [15], as well as the inhibition of osteoclast-mediated bone resorption [16]. While the advantageous effects of melatonin on osteogenesis have been extensively elucidated, its influence on vascularization remains a topic of debate [17]. Melatonin has been reported to significantly impede cell proliferation, migration, and tube formation in human umbilical vein endothelial cells (HUVECs) by down-regulating the expression of vascular endothelial growth factor (VEGFA) [18]. Thus, melatonin has been regarded as an effective antiangiogenic agent in the inhibition of tumor growth and progression [19]. Nevertheless, the antioxidant characteristic of melatonin may confer advantages to the functional integrity of vascular endothelium. Lee et al. conducted a study demonstrating that the daily administration of melatonin successfully shielded HUVECs from oxidative stress-induced mitochondrial harm, thereby restoring aortic vasorelaxation and nitric oxide release in aged mice [20]. Hence, given the significance of vascularization in the process of bone healing, it is imperative to conduct comprehensive research on the fundamental mechanisms through which melatonin governs the differentiation of endothelial cells and the interplay between osteogenesis and angiogenesis. Due to its classification as a small molecule drug, melatonin necessitates sustained release for effective local treatment of bone defects, thereby requiring its combination with biomaterials to fulfill the role of sustained release. The incorporation of a dynamic self-healing hydrogel and melatonin has been proven effectively to promote the annulus fibrous regeneration by enhancing the mitochondrial function [21]. The potential for synergizing melatonin with electrospun nanofibers to enhance periosteum-based bone regeneration is evident.

**Fig. 1:**
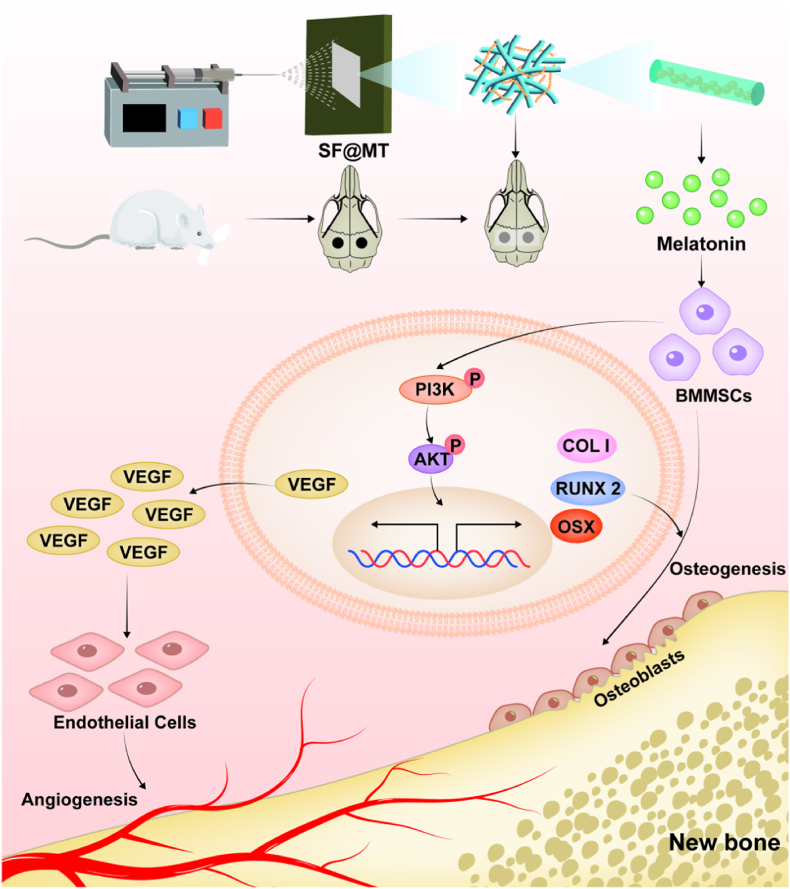
Schematic diagram of melatonin-loaded electrospun nanofibers (SF@MT) regulating the osteogenesis and angiogenesis coupling. The therapeutic efficacy of SF@MT nanofibers was evaluated in a critical-sized calvarial defect.

In the present study, we propose the hypothesis that melatonin plays a role in promoting vascularized bone regeneration by modulating the coupling of osteogenesis and angiogenesis. More specifically, apart from its positive impact on the differentiation of BMMSCs into osteoblasts, melatonin is capable of regulating the expression of the pro-angiogenic factor VEGFA in BMMSCs, thereby stimulating the formation of neovascularization. Hence, we utilized the electrospinning technique to produce a melatonin-encapsulated SF nanofibrous membrane (SF@MT). Subsequently, we assessed the impact of SF@MT nanofibers on cell proliferation, osteogenesis, angiogenesis, as well as the activation of the phosphoinositide 3-kinase/protein kinase B (PI3K/Akt) signaling pathway. Furthermore, we conducted an investigation on the therapeutic effectiveness of SF@MT nanofibers in a rat model with a critical-sized calvarial defect (Fig. 1).

## 2 Experimental section/methods

### 2.1 Fabrication of SF and SF@MT electrospun nanofibers

Initially, a total of 6 g of silkworm cocoons (Aladdin, Shanghai, China) were subjected to heating in a 3 L solution of sodium carbonate (Aladdin) with a concentration of 0.5 % (w/v) at 100 °C for 4 h. Subsequently, the cocoons were dried in an oven set at 37 °C for 12 h. The purified SF was then dissolved in a saturated lithium bromide solution (Sigma-Aldrich, St. Louis, MO, USA), and the dissolution process was carried out at 60 °C for 6 h until complete dissolution was achieved. The resulting solution was then placed into dialysis bags (MWCO 3500 Da, Aladdin) and subjected to dialysis in deionized water for 7 d.

A solution was prepared by dissolving 0.5 g of solid SF and 200 mg of melatonin (Sigma-Aldrich) in 5 mL of 1,1,1,3,3,3-hexafluoro-2-propanol (HFIP, Sigma-Aldrich). The resulting solution was then injected through a capillary tip using a 10-mL syringe. During the electrospinning process, a high voltage of 17 kV was applied to the needle, and the flow rate of the spinning solution was maintained at 2 mL/h. The SF and SF@MT electrospun nanofibers were collected on aluminum foil wrapped around the drum and subsequently dried overnight in a vacuum oven at 100 °C.

### 2.2 Characterization of SF and SF@MT electrospun nanofibers

#### 2.2.1 Scanning electron microscope (SEM)

The SF and SF@MT electrospun nanofibers underwent freeze-drying and subsequent treatment with an ion sputtering instrument (SC7620, Quorum Technologies, East Sussex, UK) to examine the surface morphology of the nanofibrous membranes an SEM (S-4800, Hitachi, Japan) operating at 15 kV.

#### 2.2.2 Contact angle tests

The nanofibrous membranes were placed on the carrier stage of a hydrophilic angle tester, and a droplet of deionized water was introduced onto the membrane. Images were captured, and the contact angles were determined using an optical goniometer (DSA25S, Oberkochen, Zeiss, Germany).

#### 2.2.3 Mechanical properties

The tensile property of the SF or SF@MT membranes was evaluated using a mechanical testing machine (Hengyi Precision Instruments Co., Shanghai, China) operating at a speed of 2.5 mm/min until rupture occurred. The MTS Testworks 4.0 software (Eden Prairie, MN, USA) was employed to calculate the slope of the stress-strain curve, which was subsequently recorded as Young's modulus.

#### 2.2.4 Degradation rate

The dried SF or SF@MT nanofibers were quantified by weighing and recorded as W_0_. They were then submerged in 5 mL of phosphate-buffered saline (PBS, Sigma-Aldrich) on a rotator at 37 °C. Following this, the lyophilized nanofibers were weighed as W_t_. The rate of degeneration was determined using the formula: remaining weight (%) = W_t_/W_0_ × 100 %.

#### 2.2.5 Release profile of melatonin from SF@MT nanofibers

The SF@MT nanofibers were immersed in 1 mL of PBS at 37 °C. The leachate was collected at specific time points. According to the standard curve of melatonin, the cumulative release profiles of melatonin were determined at the absorbance of 280 nm using a spectrophotometer (Shimadzu Co., LTD, Shanghai, China).

### 2.3 Biocompatibility and osteogenic effect of SF@MT nanofibers

#### 2.3.1 Isolation and culture of BMMSCs

BMMSCs were extracted from the femurs and tibias of euthanized 8-week-old Sprague-Dawley (SD) rats. The bone marrow cavity containing BMMSCs was flushed with alpha minimum essential medium (α-MEM, Thermo Fisher Scientific, Waltham, MA, USA), followed by the removal of red blood cells using a red blood cell lysis buffer (Beyotime, Haimen, China). BMMSCs were then cultured in α-MEM supplemented with 10 % fetal bovine serum (FBS), 100 U/ml penicillin, and 100 μg/ml streptomycin (all from Thermo Fisher Scientific) at 37 °C with 5 % CO_2_. The culture medium was changed every 3 days. The cells at passage two (P2) were used for subsequent experiments.

#### 2.3.2 Cell proliferation

The cell counting kit-8 assay (Beyotime, Haimen, China) was used to examine cell proliferation. After the BMMSCs were cultured in different electrospun nanofiber membranes, the CCK-8 solution was added to each well and incubated for 1 h at 37 °C in the dark. Subsequently, 100 μL of the solution was transferred into a 96-well plate. The optical density (OD) value was measured with a microplate spectrophotometer at 450 nm (BioTek, Winooski, VT, USA).

#### 2.3.3 Induction of osteogenic differentiation on SF@MT nanofibers

BMMSCs were seeded onto SF or SF@MT electrospun nanofibers in a 12-well plate at a density of 1 × 10^4^ cells/cm^2^. The CTRL group consisted of cells cultured on conventional tissue culture polystyrene (TCPS). To induce osteogenic differentiation, the cells were incubated in osteogenic differentiation medium consisting of Dulbecco's modified Eagle medium (DMEM) supplemented with 10 % FBS, 100 U/ml penicillin, 100 μg/ml streptomycin, 10 mM β-glycerol phosphate, 100 nM dexamethasone, and 50 μg/mL l-ascorbic acid (Sigma-Aldrich). The medium was refreshed every three days.

#### 2.3.4 Measurement of alkaline phosphatase (ALP) activity

After a 7-day period of osteogenic induction, the cells were fixed with 4 % paraformaldehyde (Sigma-Aldrich) for 30 min. Subsequently, they were exposed to ALP staining solution (Sigma-Aldrich) for 30 min in a dark environment. The stained cells were then observed under an Olympus IX51 microscope (Olympus, Tokyo, Japan).

The quantitative measurement of ALP activity was performed using an Alkaline Phosphatase S-10 Assay Kit (Beyotime), according to the manufacturerʹs protocol. Specifically, BMMSCs were dissolved in ice-cold cell lysis buffer, and following centrifugation, the resulting supernatant was incubated with a working solution for 30 min at 37 °C. The absorbance was measured at 520 nm using a microplate reader (BioTek). The protein concentration was determined using a bicinchoninic (BCA) acid protein assay kit (Beyotime), and the ALP activity was normalized to the total protein content.

#### 2.3.5 Alizarin red S (ARS) staining

Following a 21-day period of osteogenic differentiation, the cells were fixed using 4 % paraformaldehyde for 30 min. Subsequently, the cells were subjected to incubation in a 40 mM ARS solution (Sigma-Aldrich) at room temperature for 30 min. Digital images were acquired utilizing an Olympus IX51 microscope. For quantitative analysis, a 5 % perchloric acid solution (Sigma-Aldrich) was introduced to dissolve the stain present within the calcified layer, and the absorbance was measured at a wavelength of 420 nm using a PowerWave XS spectrophotometer (BioTek).

#### 2.3.6 Quantitative real-time reverse transcription–polymerase chain reaction (RT-PCR)

Total RNA was extracted using Trizol reagent (Thermo Fisher Scientific) and the complementary DNA (cDNA) was synthesized using a RevertAid First Strand cDNA Synthesis Kit (Thermo Fisher Scientific). PCR amplification was performed in a CFX96TM real-time thermal cycler (Bio-Rad, Hercules, CA, USA) using SYBR Green Supermix kit (Bio-Rad). The relative expression of gene was calculated by the 2^−ΔΔCT^ method, while glyceraldehyde-3-phosphate dehydrogenase (*Gapdh*) was used as the internal control. The primer sequences are listed in Supplementary Table 1.

#### 2.3.7 Western blot

The total protein content of BMMSCs was extracted utilizing RIPA buffer (Beyotime), and the concentration of protein was determined using a BCA protein assay kit (Beyotime). An equal quantity of protein was then separated via 10 % sodium dodecylsulfate-polyacrylamide (SDS-PAGE) gel and subsequently transferred onto a nitrocellulose membrane (Beyotime) at 4 °C. Following a 30-min incubation in a blocking buffer, the membranes were exposed to primary antibodies overnight at 4 °C. The detailed information of the primary antibodies is listed in Supplementary Table 2. The following day, the membranes were incubated with horseradish peroxidase (HRP) labeled secondary antibodies (1:20,000, ab150079, Abcam, Cambridge, UK) for 1 h at room temperature. The resulting bands were visualized using SuperSignal West Pico Substrate (Thermo Fisher Scientific) and the ChemiDoc Touch Imaging System (Bio-Rad). The intensity of the bands was quantified using Image J software (National Institutes of Health, Bethesda, MD, USA), with β-actin serving as a reference.

### 2.4 Angiogenic effect of SF@MT nanofibers

HUVECs were obtained from Procell Life Science & Technology Company (Wuhan, China). BMMSCs were cultured on either SF or SF@MT nanofibers, and the resulting conditioned mediums were collected after 7 days of osteogenic induction. Growth factor-reduced Matrigel (BD Biosciences, San Jose, CA, USA) was prepared following the manufacturer's instructions. To be specific, 60 μL of Matrigel was introduced into individual wells of a 96-well plate and allowed to incubate at 37 °C for 30 min. HUVECs were then seeded at a density of 1000 cells per well and then exposed to the following groups of substances: leachate from SF nanofibers, leachate from SF@MT nanofibers, conditioned medium from BMMSCs cultured on SF nanofibers, and conditioned medium from BMMSCs cultured on SF@MT nanofibers. Following a 6-h incubation period, the formation of endothelial tubes was observed using an Olympus IX51 microscope. For quantitative analysis, the number of tubes was determined using the Image J software.

### 2.5 Inhibition of PI3K assay

To suppress PI3K activity, BMMSCs were seeded in a 6-well plate at a density of 1 × 10^6^ cells/well. The cells were exposed to 10 μM LY294002, a specific inhibitor of PI3K, while the control (CTRL) group received an equivalent volume of dimethyl sulfoxide (DMSO, Sigma-Aldrich).

### 2.6 In vivo evaluation of SF@MT nanofibers on bone regeneration

A total of thirty male SD rats (aged 8–10 weeks and weighing 200–300 g) were procured from the Experimental Animal Center of Soochow University. Ethical approval was obtained from the Ethics Committee of Soochow University (No.SUDA20230428A02) for all animal experiments. The rats were housed under controlled conditions, including constant humidity (50–60 %), temperature (22–24 °C), and a light cycle from 6 a.m. to 6 p.m.

#### 2.6.1 Establishment of a rat calvarial critical-sized defect model

Rats were anesthetized using intraperitoneal injection of 3 % sodium pentobarbital (Shanghai Merck Co., Ltd., Shanghai, China) at a dosage of 1.5 mL/kg body weight. Following complete shaving and sterilization, a longitudinal midline incision was performed on the scalp, and the soft tissue was meticulously separated to expose the calvarium. Two circular defects measuring 5 mm in diameter were then created in the calvarium, and the bone fragments were carefully removed. The defect area was then covered with either SF or SF@MT nanofibrous membranes, while rats in the Defect group were left untreated. Postoperatively, penicillin at a dosage of 6000 units was administered once daily for three consecutive days.

#### 2.6.2 Micro-computed tomography (μCT) scanning

Calvarial specimens were collected and preserved in 10 % formalin (Sigma-Aldrich) at 4 and 8 weeks after surgery. To assess bone regeneration in the defect areas, a high-resolution μCT (SkyScan 1176, Bruker Corporation, Billerica, MA, USA) was employed with the following parameters: 65 kV, 385 mA, and 1 mm Al filter. The three-dimensional (3D) reconstruction was performed using Mimics Research software (Materialise, Leuven, Belgium). The region of interest (ROI) reconstruction area for the defect consisted of a consistent segment centered on the calvarium. CT Analyzer software (Bruker) was used to calculate bone volume ratio (BV/TV, %), trabecular thickness (Tb.Th., μm), and bone mineral density (BMD, g/cm^3^) for evaluating bone microstructure.

#### 2.6.3 Histological analysis

The harvested specimens underwent decalcification using a 10 % ethylene diamine tetraacetic acid (EDTA, Sigma-Aldrich) solution for 4 weeks, followed by embedding in paraffin blocks. The specimens were cut into sections with a thickness of 5 μm. For hematoxylin and eosin (H&E) staining, the sections were stained with hematoxylin for 3 min, followed by eosin solution for 1 min (all from Jiancheng, Nanjing, China). For Masson's trichrome staining, the sections were stained with a solution containing hematoxylin and lichun red acid for 5 min each (Solaibao Technology Co., Ltd, Beijing, China). Following the processes of dehydration and sealing, digital images were captured using a bright field microscope (Zeiss).

#### 2.6.4 Immunohistochemical (IHC) staining

For IHC staining, the tissue sections were subjected to deparaffinization using xylene, followed by hydration using graded ethanol. To inhibit endogenous peroxidase activity, the sections were then incubated in a 3 % hydrogen peroxide solution (Sigma-Aldrich). Additionally, the sections were treated with 2 mg/mL of testicular hyaluronidase (Sigma-Aldrich) for 30 min at 37 °C. Subsequently, the sections were blocked using 1.5 % goat serum and incubated with specific primary antibodies overnight at 4 °C, including anti-COL1A1 (1:500, ab138492, Abcam), anti-osteocalcin (1:500, ab93876, Abcam), anti-CD31 (1:500, A0378, ABclonal, Wuhan, China), and anti-VEGFA (1:500, A12303, ABclonal). The following day, the sections were incubated with biotinylated goat anti-rabbit secondary antibodies for a duration of 30 min. Signal amplification was achieved using the Vectastain Elite ABC kit. The staining process involved the use of a 3,3′-diaminobenzidine (DAB) solution (all from Vector Laboratories, Burlingame, CA, USA), and counterstaining of the nuclei was performed using hematoxylin. Images of 5 randomly chosen fields in each group were taken with a bright field microscope. The Image J software (NIH, Bethesda, MD, USA) was used to analyze the average gray values to obtain immunohistochemical staining quantitative results.

### 2.7 Statistical analysis

Statistical analyses were performed using GraphPad Prism 9.2 software (GraphPad Software Inc., San Diego, CA, USA). All data were expressed as the mean ± standard deviation. The two-tailed Student's t-test was used to compare the two groups, and one-way analysis of variance (ANOVA) was used to discern the significant differences among multiple groups. *P* values < 0.05 (*) or < 0.01 (**) were considered as statistically significant.

## 3 Results

### 3.1 Characterization of SF@MT electrospun nanofibers

**Fig. 2:**
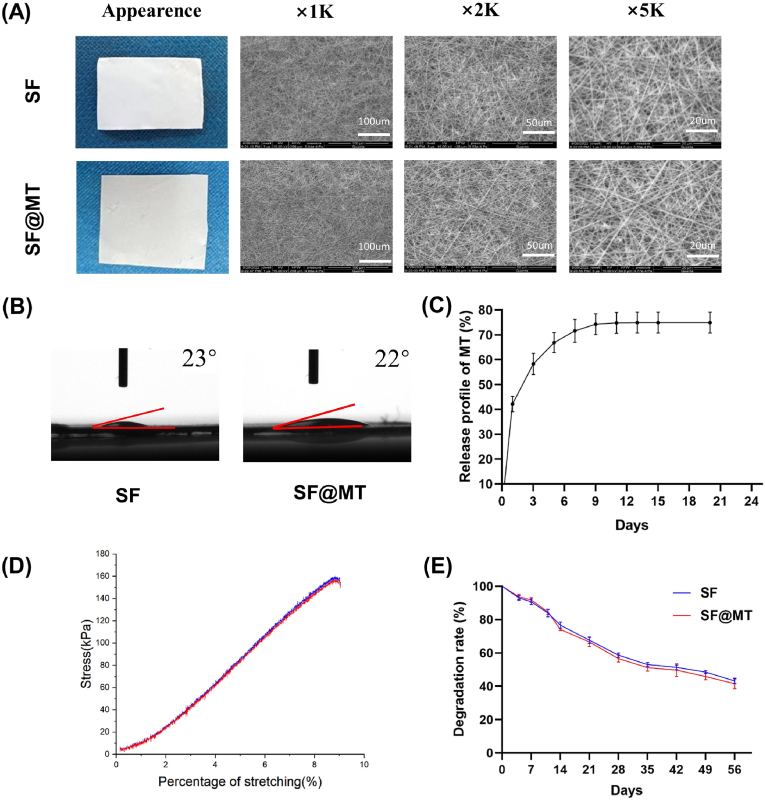
Morphology and characterization of SF and SF@MT electrospun nanofibers. (A) Gross appearance and SEM micrographs of the SF and SF@MT electrospun nanofibers. (B) Water contact angle measurements determined the hydrophilicity of SF and SF@MT nanofibers. (E) Release profile of melatonin from SF@MT nanofibers, n = 3. (D) Tensile strength of SF and SF@MT nanofibers. (E) Degradation rate of SF and SF@MT nanofibers, n = 3. Data are presented as means ± SD.

The electrospinning technique was employed to fabricate SF and SF@MT nanofibrous membranes (Fig. 2A). SEM analysis demonstrated that the membranes exhibited a homogeneous distribution of electrospun nanofibers with random orientation. Notably, there was no discernible disparity in surface morphology between the SF and SF@MT nanofibers. The contact angles of the SF and SF@MT nanofibers were measured to be 23° and 22°, respectively, indicating the desirable hydrophilic nature of both nanofibrous membranes (Fig. 2B). The release profile analysis of SF@MT nanofibers exhibited an initial rapid release of melatonin on the first day (approximately 42 %), followed by a comparatively slower release until day 14, indicating a sustained release behavior of SF@MT nanofibers (Fig. 2C). The stress-strain experiments demonstrated that the tensile strength of SF and SF@MT nanofibrous membranes was measured to be 0.69 ± 0.16 and 0.66 ± 0.04 MPa, respectively (Fig. 2D). After immersing in PBS for eight weeks *in vitro*, the degradation rates of SF and SF@MT nanofibers were determined to be 41.5 ± 4.2 % and 43.2 ± 1.0 %, respectively (Fig. 2E).

### 3.2 SF@MT nanofibers promote the osteogenic differentiation of BMMSCs

**Fig. 3:**
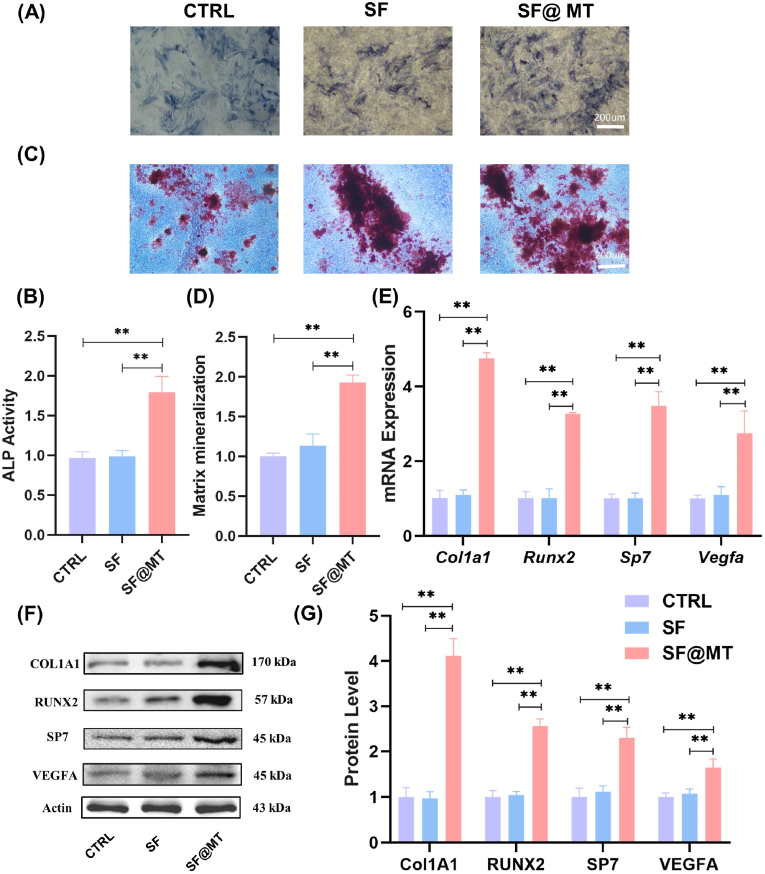
Eﬀect of SF@MT electrospun nanofibers on the osteogenic differentiation of BMMSCs. BMMSCs were seeded on SF and SF@MT nanofibers and induced toward the osteogenic differentiation, while cells cultured on the conventional tissue culture polystyrene (TCPS) served as the control (CTRL) group. (A) ALP staining was performed after 7 days of induction. (B) Quantitative analysis of ALP activity, n = 3. (C) Alizarin Red S (ARS) staining was performed after 21 days of induction. (D) Quantification of the stained mineral layers indicated that SF@MT nanofibers promoted matrix mineralization, n = 3. (E) The gene expression of osteogenic makers, including *Col1a1*, *Runx2*, *Osterix*, and *Vegfa*, was determined by RT-PCR, n = 4. (F–G) The protein levels of COL Ⅰ, RUNX2, SP7, and VEGFA were examined by Western blot, n = 3. Data are presented as means ± SD. Statistically significant differences are indicated by * where *P* < 0.05 or ** where *P* < 0.01 between the indicated groups. (For interpretation of the references to color in this figure legend, the reader is referred to the Web version of this article.)

BMMSCs cultured on SF and SF@MT electrospun nanofibers exhibited comparable and well-spread cell morphology (Suppl. Fig. 1). The live/dead cell staining revealed a substantial number of viable cells with minimal presence of deceased cells, suggesting that both SF and SF@MT nanofibers have favorable biocompatibility (Suppl. Fig. 2-4). After a 7-day period of osteogenic induction, the SF@MT group exhibited a more pronounced staining of ALP, a recognized marker for early osteogenic differentiation, potentially attributable to the sustained release of melatonin (Fig. 3A). Quantitative analysis substantiated that the ALP activity in the SF@MT group surpassed that in the control (CTRL) and SF groups by 79.2 % and 81.8 %, respectively (Fig. 3B). Subsequent to a 21-day interval, BMMSCs cultured on SF@MT nanofibers demonstrated a robust positive response to Alizarin Red S staining, indicative of a heightened level of matrix mineralization, a crucial indicator for late osteogenesis (Fig. 3C). The results of quantitative analysis indicate that the SF@MT group exhibited a calcium deposition level that was 110.0 % higher than the CTRL group and 106.4 % higher than the SF group (Fig. 3D).

Furthermore, the real-time RT-PCR findings suggest that the gene expression of osteogenic markers was up-regulated in BMMSCs cultured on SF@MT nanofibers. For instance, the transcript levels of *Col1a1*, *Runx2*, and *Sp7* were significantly increased by 333.7 %, 224.0 %, and 244.7 %, respectively, in comparison to the SF group (Fig. 3E). Specifically, the mRNA level of *Vegfa* in the SF@MT group exhibited a significant increase of 175.3 % and 150.8 % compared to the CTRL and SF groups, respectively, suggesting that the utilization of SF@MT nanofibers holds promise for inducing angiogenesis. This assertion was further supported by Western blot analysis, which revealed a substantial up-regulation of COL1A1, RUNX2, SP7, and VEGFA protein expression in the SF@MT group, with respective increases of 325.4 %, 145.0 %, 106.3 %, and 52.9 % compared to the SF group (Fig. 3F and G). These findings demonstrate the effective enhancement of osteogenic differentiation in BMMSCs and the concurrent elevation of VEGFA expression facilitated by SF@MT electrospun nanofibers. Immunofluorescence staining confirmed the strongly positive staining for COL1A1 (Suppl. Fig. 5) and VEGFA expression (Suppl. Fig. 6) in the SF@MT group.

### 3.3 SF@MT nanofibers regulates the osteogenesis–angiogenesis coupling

**Fig. 4:**
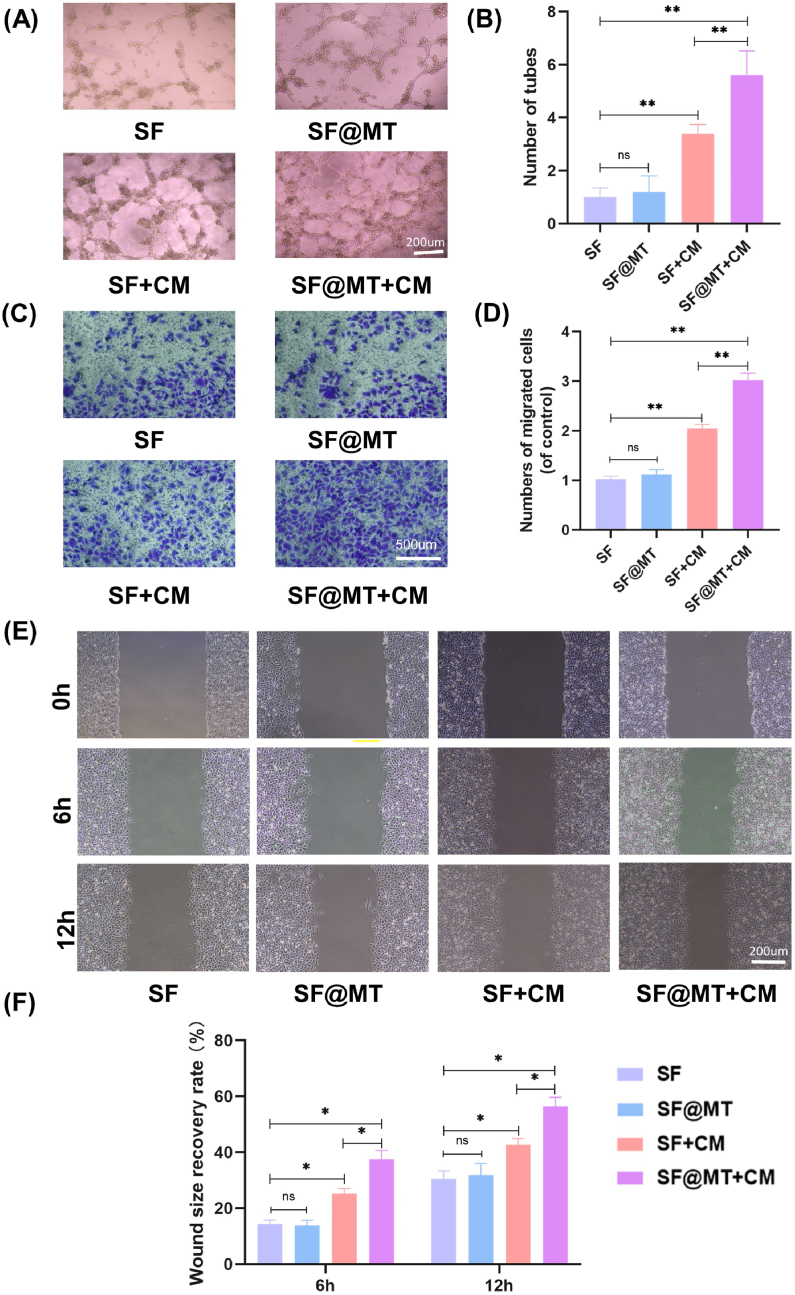
The regulation of osteogenesis and angiogenesis coupling by the conditional medium derived from BMMSCs cultured on SF@MT nanofibers. HUVECs were treated with SF and SF@MT-extracted leachate and the conditioned medium derived from differentiated BMMSCs on the two nanofibers (SF + CM and SF@MT + CM). (A) Representative images of endothelial tube formation. Scale bar = 200 μm. (B) Quantitation of the numbers of endothelial tubes, n = 3. (C) Representative images of transwell cell migration of HUVECs. Scale bar = 200 μm. (D) Quantitation of cell migration capacity, n = 3. (E) Representative images of scratch wound assay of HUVECs. Scale bar = 200 μm. (F) Quantitation of scratch wound assay, n = 3. Data are presented as means ± SD. Statistically significant differences are indicated by * where *P* < 0.05 or ** where *P* < 0.01 between the indicated groups. ns, no significant difference. CM, conditioned mediums.

In order to investigate the impact of SF@MT nanofibers on angiogenesis, HUVECs were exposed to SF and SF@MT-extracted leachate, as well as the conditioned medium derived from differentiated BMMSCs on the two types of nanofibers (SF + CM and SF@MT + CM, respectively). There was no statistically significant difference in the ability to stimulate tube formation between the SF and SF@MT-extracted leachate. However, when HUVECs were treated with the conditioned medium, a significant increase in endothelial tube formation was observed in both the SF-CM and SF@MT-CM groups compared to the SF and SF@MT leachate groups (Fig. 4A). The quantitative analysis demonstrated a significant increase in the number of endothelial tubes in the SF@MT-CM group compared to the SF@MT and SF-CM groups, with a respective increase of 475.0 % and 91.7 % (Fig. 4B). The transwell assays indicated that both SF-CM and SF@MT-CM significantly enhanced the cell migration capacity of HUVECs, while the leachate from SF and SF@MT had minimal impact (Fig. 4C and D). The results of the scratch wound assay exhibited a similar trend, with HUVECs in the SF@MT-CM group demonstrating a 35.8 % and 24.9 % faster migration rate compared to the SF@MT and SF-CM groups, respectively (Fig. 4E and F). These findings suggest that SF or SF@MT nanofibers alone did not have a direct stimulatory effect on the angiogenesis of HUVECs. However, the use of SF-CM and SF@MT-CM significantly enhanced the ability of HUVECs to form tubes and migrate. The observed regulation of osteogenesis and angiogenesis coupling may be attributed to the melatonin-mediated expression of VEGFA in differentiated BMMSCs cultured on SF@MT nanofibers.

### 3.4 SF@MT regulates the osteogenesis and angiogenesis coupling via the PI3K-AKT signaling pathway

**Fig. 5:**
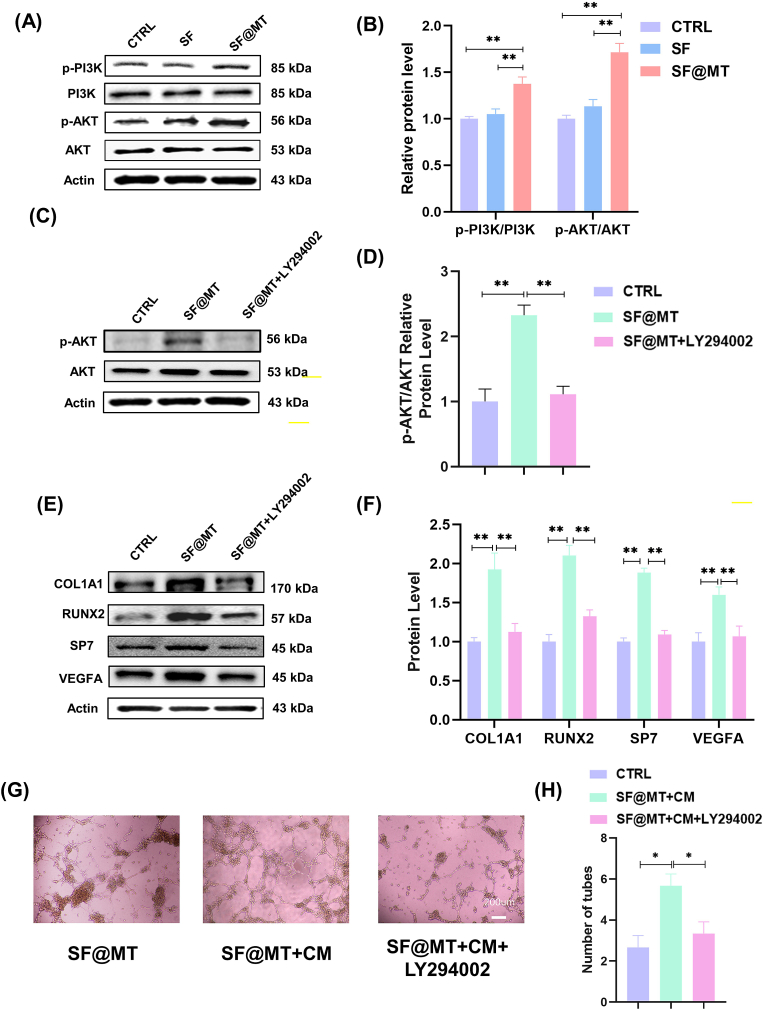
Inhibition of PI3K/AKT by LY294002 abolished the effect of SF@MT nanofibers on the osteogenesis of BMMSCs and related angiogenesis. (A) The protein levels of p-PI3K, PI3K, *p*-AKT, and AKT were measured by Western blot. (B) Quantification of the phosphorylation levels of PI3K and AKT, n = 3. (C) The effect of LY294002 on the phosphorylation of AKT in SF@MT-cultured BMMSCs was evaluated by Western blot. (D) Quantification of *p*-AKT levels in LY294002-treated BMMSCs, n = 3. (E–F) The protein levels of COL Ⅰ, RUNX2, SP7, and VEGFA were examined by Western blot, n = 3. (G) The effect of LY294002 on the angiogenesis of HUVECs was investigated by endothelial tube formation assays. Scale bar = 200 μm. (H) Quantitation of the numbers of endothelial tubes, n = 3. Data are presented as means ± SD. Statistically significant differences are indicated by * where *P* < 0.05 or ** where *P* < 0.01 between the indicated groups.

We further investigated the mechanisms underlying the coupling of osteogenesis and angiogenesis mediated by melatonin. Western blot experiments demonstrated a notable increase in the expression of p-PI3K and *p*-AKT in BMMSCs cultured on SF@MT nanofibers (Fig. 5A). Specifically, the phosphorylation levels of PI3K and AKT in the SF@MT group exhibited a significant rise of 31.1 % and 51.2 %, respectively, compared to the SF group, indicating the activation of the PI3K-AKT pathways (Fig. 5B). In order to investigate the role of PI3K/Akt in the regulation of osteogenesis and angiogenesis coupling, BMMSCs were subjected to treatment with the PI3K specific inhibitor LY294002. The results of Western blot assays provided confirmation that the administration of LY294002 led to a significant reduction in the phosphorylation level of AKT by 52.2 % in BMMSCs cultured in SF@MT (Fig. 5C and D). The inhibition of PI3K/AKT resulted in the nullification of the osteogenic impact of SF@MT nanofibers on BMMSCs, as demonstrated by the decrease in matrix mineralization (Suppl. Fig. 7) and the down-regulation of osteoblast-specific marker expression (Fig. 5E and F, Suppl. Fig. 8). Specifically, the treatment with LY294002 resulted in a decrease of 41.7 % and 32.9 % in the transcript and protein levels of COL1A1, respectively, in comparison to the SF@MT group. The expression of VEGFA in LY294002-treated BMMSCs exhibited a statistically significant reduction of 22.7 % at the mRNA level and 33.2 % at the protein level, when compared to the SF@MT group. Additionally, the administration of LY294002 led to a notable decrease of 41.2 % in the tube formation ability of HUVECs (Fig. 5G–H), indicating the crucial involvement of the PI3K/AKT signaling pathway in the synergistic enhancement of osteogenesis and angiogenesis facilitated by SF@MT.

### 3.5 Implantation of SF@MT nanofibers repaired rat calvarial defects

**Fig. 6:**
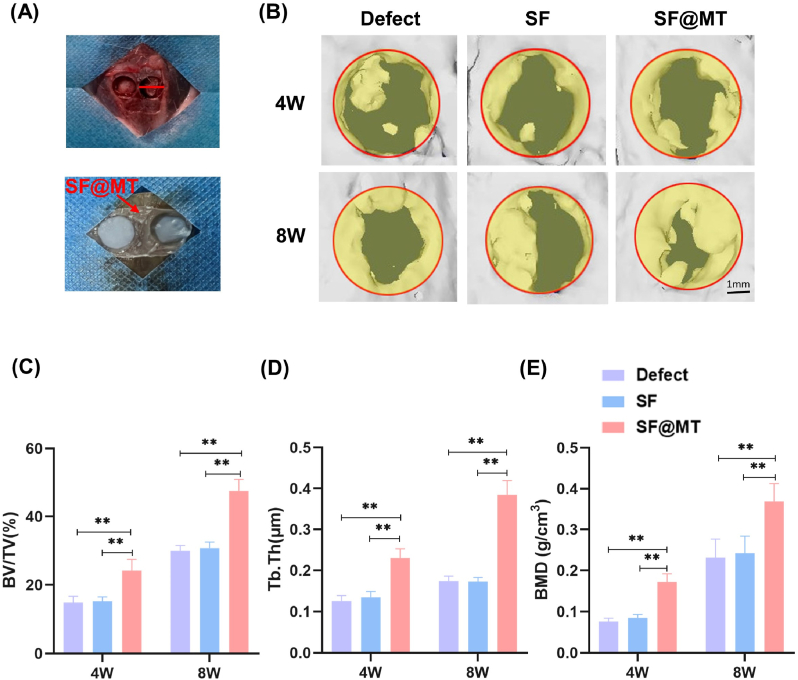
Micro-CT evaluation of *in vivo* calvarial bone regeneration. (A) Critical-sized defects with 5 mm in diameter were created on rat calvarium and subsequently covered with SF and SF@MT nanofibrous membranes, while the Defect group was left untreated. (B) The new bone formation in the calvarial defects was evaluated by micro-CT imaging and 3D reconstruction at 4 and 8 weeks post-surgery. (C–E) Quantitative analysis of BV/TV, Tb.Th and BMD in the defect area after 4 and 8 weeks of implantation, n = 5. Data are presented as means ± SD. Statistically significant differences are indicated by * where *P* < 0.05 or ** where *P* < 0.01 between the indicated groups.

In order to evaluate the therapeutic effectiveness of SF@MT nanofibers, critical-sized defects measuring 5 mm in diameter on the rat calvarium were established (Fig. 6A). Micro-CT was employed at 4 and 8 weeks after the surgery to assess the regeneration of the calvarial bone. The 3D reconstruction images clearly demonstrate that the SF@MT group displayed superior bone regeneration in comparison to both the Defect and SF groups. This is supported by the observation of new bone formation along the edges of the defects (Fig. 6B). Quantitative analysis indicate that the implantation of SF@MT nanofibers led to significant enhancements in bone microarchitecture and quality, as evidenced by increases in calculate BV/TV, Tb.Th., and BMD. However, there was no significant difference observed between the Defect and SF groups. Specifically, the BV/TV values in the SF@MT group were 59.1 % (at 4 weeks post-surgery) and 54.7 % (at 8 weeks post-surgery) higher than those in the SF group (Fig. 6C). Similarly, the implantation of SF@MT nanofibers resulted in a 70.5 % improvement in Tb.Th (Fig. 6D). and a 122.7 % improvement in BMD (Fig. 6E) compared to the SF group at 8 weeks post-surgery.

**Fig. 7:**
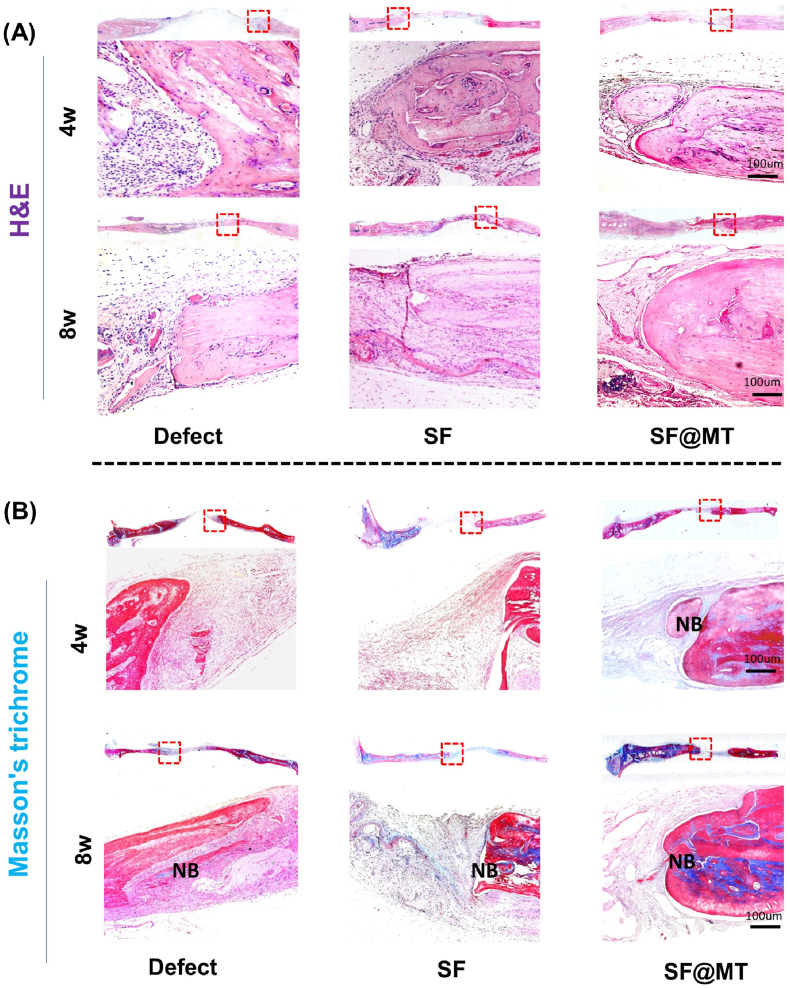
Histological analysis of the newly formed bone tissue in the calvarial defects at 4 and 8 weeks post-surgery. (A) Representative hematoxylin and eosin (H&E) staining of the defect area implanted with SF or SF@MT nanofibers. (B) Representative Masson's trichrome staining of collagen deposition. Scale bar = 100 μm. NB: new bone.

Histological analysis was performed to further assess the bone healing process in the calvarial defects. H&E staining demonstrated that, after a 4-week period, both the Defect group and SF group showed minimal bone repair. In contrast, the implantation of SF@MT nanofibers facilitated a significant formation of new bone tissues, as indicated by the presence of a moderate amount of calcium nodule deposition at the defect site (Fig. 7A). Additionally, Masson's trichrome staining revealed a substantial accumulation of collagen in the SF@MT group at 4 weeks post-surgery, suggesting that SF@MT expedited the process of bone healing (Fig. 7B). Consistently, trabecular bone repair was consistently observed in the SF@MT group after 8 weeks of implantation, while inadequate bone repair was observed in the Defect and SF groups.

**Fig. 8:**
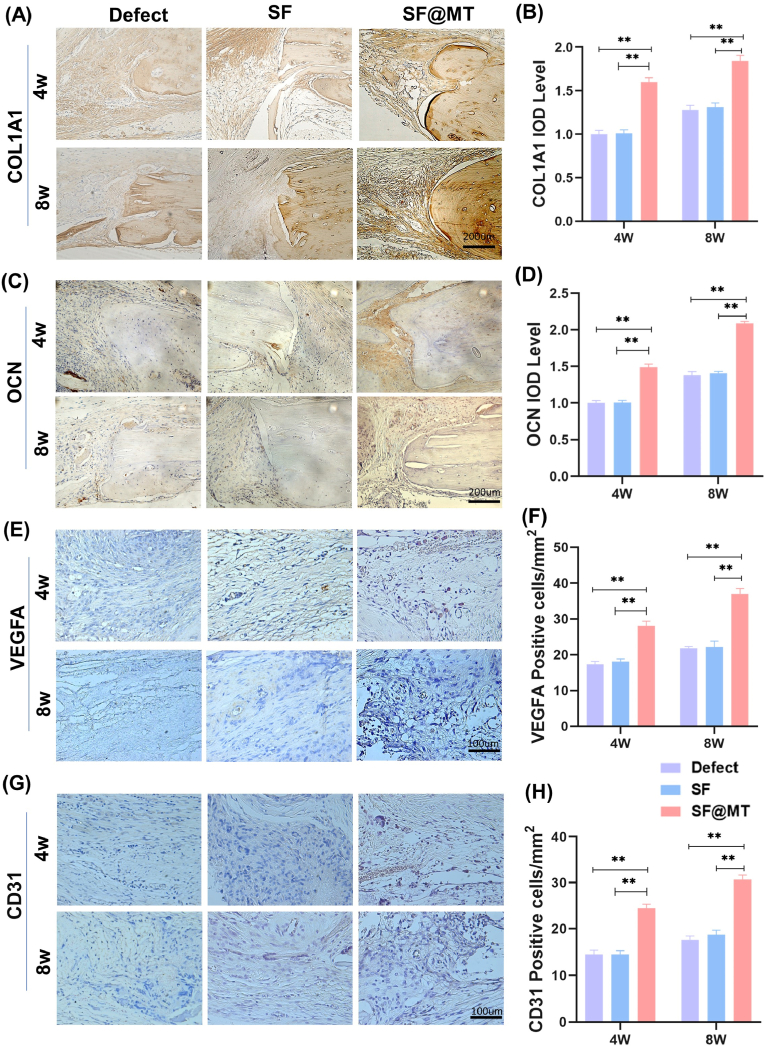
Immunohistochemical analysis of the newly formed bone tissue and blood vessels in the calvarial defects. (A) Representative images of immunofluorescence staining for type I collagen (COL1A1) in the defect area. Scale bar = 200 μm. (B) Quantification of the COL I-positive area at 4 and 8 weeks post-surgery, n = 3.(C) Representative images of immunofluorescence staining for Osteocalcin (OCN) in the defect area. Scale bar = 200 μm. (D) Quantification of the OCN-positive area at 4 and 8 weeks post-surgery, n = 3. (E) Representative images of immunofluorescence staining for vascular endothelial growth factor α (VEGFA) in the defect area. Scale bar = 200 μm. (F) Quantification of the VEGFA-positive area at 4 and 8 weeks post-surgery, n = 3. (G) Representative images of immunofluorescence staining for CD31^+^ cells that represent the newly formed blood vessels. Scale bar = 200 μm. (H) Quantification of the CD31-positive vessels at 4 and 8 weeks post-surgery, n = 3. Data are presented as means ± SD. Statistically significant differences are indicated by * where *P* < 0.05 or ** where *P* < 0.01 between the indicated groups.

Immunohistochemical staining revealed extensive positive staining for COL1A1 and osteocalcin (OCN) in the SF@MT group at 4 and 8 weeks post-surgery, indicating the robust synthesis of bone matrix at the defect site (Fig. 8A and C). Compared to the defect group, the expression level of COL1A1 and OCN was significantly increased by 43.7 % and 51.6 % (at 8 weeks post-surgery) following SF@MT nanofiber implantation (Fig. 8B and D). In order to assess the impact of SF@MT nanofibers on angiogenesis, immunohistochemical staining was conducted to examine the presence of VEGFA and CD31. The findings demonstrated a robust positive staining for VEGFA (Fig. 8E) and CD31 (Fig. 8G) in the SF@MT group, in contrast to the limited staining observed near the defect site in both the Defect and SF groups. Quantitative analysis revealed that the expression level of VEGFA in the SF@MT group was 61.9 % (at 4 weeks post-surgery) and 69.6 % (at 8 weeks post-surgery) higher compared to that in the Defect group (Fig. 8F). Consistently, the SF@MT group exhibited a 68.8 % increase in CD31^+^ cell percentages at 4 weeks post-surgery and a 74.6 % increase at 8 weeks post-surgery compared to the Defect group. These findings indicate a significant formation of new blood vessels (Fig. 8H). These results provide evidence that the implantation of SF@MT nanofibers promotes the generation of new osseous tissue and the reestablishment of new capillaries, thereby facilitating the repair of critical-sized bone defects.

## 4 Discussion

The periosteum is a crucial component in the process of bone regeneration, serving as a reservoir for storing local growth factors and other bioactive molecules. It plays a vital role in providing or recruiting skeletal stem/progenitor cells for the formation of new bone. In comparison to bone-marrow stromal cells, the skeletal stem cells derived from the periosteum have demonstrated significantly greater efficacy in facilitating endochondral ossification during the regeneration of bone [22]. In the process of traction osteogenesis, new bone is mainly generated by intramembrane osteogenesis, that is, MSCs differentiate into osteoblasts, secrete bone matrix, and finally mineralize into bone. Traction osteogenesis is also affected by the absence of periosteum, because abundant blood vessels in periosteum provides the necessary cellular support sufficient nutrients for new bone formation [23]. As a result, the development of artificial and biomimetic periosteum has been pursued to replicate the biological function of the native periosteum, with the aim of recruiting skeletal stem cells and restoring the structural integrity of bone tissue. Zhang et al. developed an artificial hydrogel periosteum by combining methacrylamide gelatin (GelMA), E7 peptide, and Wharton's jelly, which exhibited favorable mechanical properties and enhanced osteoinductive function. This periosteum effectively recruited BMMSC chemotaxis, leading to improved efficiency in fracture repair [24]. The SF electrospun nanofibers in this study are a naturally derived material that can undergo complete degradation *in vivo*. The inclusion of melatonin in the SF nanofiber membranes presents significant advantages compared to previous periosteal constructs created using synthetic polymer materials [25,26]. Unlike artificial periosteum that relies on osteogenic growth factors like BMP-2 and VEGFA, the endogenous melatonin not only has the capacity to simultaneously stimulate osteogenesis and angiogenesis but also possesses translational potential due to its biosafety [27]. Consequently, the melatonin-encapsulated bionic periosteum with adjustable shape offers a regenerative microenvironment that facilitates bone healing.

Given its distinctive membrane-like structure and suitable physical and chemical properties, electrospun fibers are commonly employed as dressings for wound healing. Nezamoleslami et al. utilized electrospinning to fabricated sandwich-structured multilayered mats and demonstrated that the controlled release of ceftazidime from the gelatin layer within the mats exhibited potent antibacterial activity without negatively affecting the viability of fibroblast cells [28]. In order to facilitate bone regeneration, a multilayer electrospun nanofibrous membrane was designed, capable of sequentially releasing MT01 (a single-stranded cytosine-phosphate-guanosine oligodeoxynucleotide) and stromal cell-derived factor 1α (SDF-1α) from the inner and outer layers, respectively. The implantation of a dual-layer structure membrane in a rat calvarial bone defect was found to be effective in promoting bone regeneration by stimulating the recruitment of stem cells in the early phase and promoting osteogenic differentiation in the late phase [29]. In this study, SF electrospun membranes with nanoscaled fibers were fabricated, allowing for sustained release of melatonin from the nanofibers. *In vitro* culturing of BMMSCs on SF@MT nanofibers resulted in a significant improvement in their osteogenic differentiation, as evidenced by the high level of ALP activity, increased calcium deposition, and up-regulation of osteoblast-specific marker genes. In accordance with our findings, the incorporation of melatonin into poly(lactic-*co*-glycolic acid) electrospun nanofibers demonstrated the ability to induce osteogenesis through the activation of the Wnt/β-catenin signaling pathway, leading to enhanced osteointegration at the titanium-bone interface, even in mice with type 1 diabetes mellitus [30]. However, it should be noted that electrospun nanofiber membranes suffer from a lack of mechanical support. In order to overcome this drawback, Li et al. have effectively devised a three-dimensional sponge scaffold utilizing electrospun nanofibers, which has been demonstrated to possess robust mechanical support and is deemed suitable for the treatment of substantial bone defects [31]. Hence, the utilization of electrospun nanofibrous membranes or scaffolds as a viable approach to attain targeted and uninterrupted melatonin release holds potential for serving as a promising artificial periosteum.

The principal discovery in this investigation lies in the ability of SF@MT nanofibers to stimulate vascularization within recently developed cortical bone, as indicated by the robust positive immunohistochemical staining for CD31, a recognized marker for capillaries. Intriguingly, the application of the leachate derived from SF@MT nanofibers in *in vitro* treatment had minimal impact on the migratory and tube formation abilities of HUVECs, aligning with prior research that demonstrated the suppressive influence of melatonin on endothelial cell angiogenesis [32]. Conversely, when HUVECs were exposed to the osteogenic conditioned medium obtained from SF@MT-cultured BMMSCs, a noteworthy enhancement in angiogenesis was observed, indicating that melatonin modulates angiogenesis in a manner contingent upon BMMSCs. According to a recent study, the administration of melatonin has been found to enhance angiogenesis mediated by BMMSCs by increasing the levels of VEGFA and expedite the repair of tibia bone defects in rats with osteoporosis [33]. Further analysis indicated that SF@MT nanofibers stimulated the expression of VEGFA, a pro-angiogenic factor, in differentiated BMMSCs. Similarly, Al-Otaibi et al. confirmed that the transplantation of BMMSCs pre-treated with melatonin, as opposed to BMMSC monotherapy, promoted wound healing in rat skin while reducing collagen deposition. This effect may be attributed to the up-regulation of VEGFA in melatonin-treated BMMSCs [34]. Furthermore, alongside VEGFA, platelet-derived growth factor-BB (PDGF-BB) serves as a significant regulator in the coordination of angiogenesis and osteogenesis. The introduction of exogenous PDGF-BB to mice subjected to ovariectomy resulted in augmented trabecular and cortical bone mass, while also inducing the development of a distinct type H endothelium that exhibited a robust positive expression of CD31 and endomucin [35]. Li et al. devised an electrospun scaffold that was modified with a leptin receptor antibody, tailored to accommodate various phases of bone healing. Following the implantation of the synthetic periosteum into the cranial defect, the subsequent bone formation revealed the emergence of osteogenesis-coupled type H capillaries. This occurrence is potentially attributed to the secretion of PDGF-BB by the recruited skeletal stem cells [36]. While the precise influence of melatonin on the development of type H vessels remains uncertain, forthcoming investigations will comprehensively explore the underlying mechanism of melatonin-mediated angiogenesis in conjunction with osteogenesis.

Previous studies have demonstrated that the osteogenic differentiation of BMMSCs can be promoted through parocrine cytokines secreted by endothelial cells [37]. In particular, Li et al. showed that exosomes secreted from human microvascular endothelial cells improved the osteogenic differentiation of BMMSCs while inhibiting the adipogenic differentiation. Molecular experiments revealed that exosomal miR-5p-72106_14 was involved in the regulation of the stem cell fate by inhibiting signal transducers and activators of transcription 1 (STAT1) [38]. Furthermore, the process of bone regeneration is intricately intertwined with the provision of nutrients, as angiogenesis within the bone facilitates the delivery of vital nutrients necessary for the regeneration and repair of bone tissue, while simultaneously removing metabolic waste [39].

The present study aimed to further investigate the involvement of the PI3K/Akt signaling pathway in the process of osteogenesis-mediated vascularization. It was observed that culturing BMMSCs on SF@MT nanofibers resulted in a significant increase in the phosphorylation levels of PI3K and AKT. Conversely, inhibition of PI3K activity using LY294002 led to a down-regulation of osteogenic markers and VAEGFA expression. The angiogenesis assay provided additional evidence, showing that HUVECs lost their ability to form vascular tubes when exposed to the conditioned medium from LY294002-treated BMMSCs. These findings highlight the critical role of the PI3K/AKT pathway in regulating the interplay between osteogenesis and angiogenesis. In accordance with our findings, Ren et al. observed the activation of the PI3K/AKT signaling pathway in the melatonin-induced preservation of the osteogenic potential of MC3T3-E1 preosteoblast cells against iron overload [40]. Conversely, the inhibition of PI3K phosphorylation by LY294002 hindered the osteointegration of titanium implant surfaces treated with innovative non-thermal atmospheric plasma, leading to a decline in osteoblast proliferation and differentiation [41]. The participation of the PI3K/AKT pathway has been previously validated in a study where the inhibition of PI3K resulted in the elimination of the bone regenerative impact of nano-laponite hydrogel, as demonstrated by the attenuation of calcified matrix formation. Furthermore, Jia et al. discovered that the transplantation of BMMSCs effectively mitigated adenine-induced injuries to renal tissues and enhanced the density of glomerular capillaries by enhancing VEGFA-induced angiogenesis through the activation of the PI3K/Akt signaling pathway [42]. In our future investigations, we will continue to elucidate the involvement of PI3K and Akt in the process of angiogenesis mediated by BMMSCs.

It is important to note that this study has a limitation in that it did not consider the regulation of inflammatory immunity in the design of the SF@MT artificial periosteum. To modulate the inflammatory response, an injectable periosteal matrix-derived hydrogel has been reported that promotes the recruitment of macrophages and facilitates the transition from M1 to M2 phenotypes during the initial stages of bone regeneration [43]. As well, melatonin has been shown to regulate circadian rhythms by participating in a variety of multifaceted intracellular signaling networks. The central circadian clock regulates endochondral bone formation, as evidenced by fast DNA replication in the daytime while matrix synthesis at night. The underlying mechanism involves activation of melatonin receptor 1 (MTR1) that periodically leads to the AMPKβ1 phosphorylation and induces the expression of the core clock molecule brain and muscle Arnt-like protein-1 (BMAL1) [44]. Subsequent investigations will prioritize the examination of melatonin's influence on circadian rhythm and inflammatory immunity.

Additionally, the absence of personalized design for the artificial periosteum represents an additional constraint in the current investigation. Tissue engineered periosteum has been successfully employed to mimic the bone healing process mediated by the periosteum [45]. The implantation of a modified tissue engineered periosteum allograft, which exhibited responsiveness to matrix metalloproteinase, has demonstrated superior efficacy in promoting localized vascularization and endochondral ossification of the graft. This is achieved by facilitating early stage recruitment of endothelial cells, formation of vasculature, and integration with host vessels and nerves [46]. Despite the observed promotion of bone regeneration in normal rat calvarial defects by melatonin-loaded nanofibers, it is imperative to administer higher dosages of melatonin to enhance the osteogenic differentiation and matrix mineralization of BMMSCs derived from osteoporotic rats [47]. The development of a controllable melatonin release system that can effectively and adaptively operate in diverse clinical application scenarios becomes crucial. Henceforth, forthcoming research endeavors will prioritize the application of tissue engineered periosteum for the purpose of coordinating effective bone regeneration processes, including the recruitment of skeletal stem cells, modulation of the inflammatory microenvironment, stimulation of local vascularization, and mineralization of the newly synthesized bone matrix.

## 5 Conclusions

In this study, we have achieved successful development of a membrane-like artificial periosteum by encapsulating melatonin within SF electrospun nanofibers. The SF@MT nanofibrous membrane has demonstrated exceptional flexibility, suitable mechanical stability, and favorable biocompatibility. The sustained release of melatonin resulted in the enhanced osteogenic differentiation of BMMSCs and upregulation of the pro-angiogenic factor VEGFA expression at both the transcript and protein levels in BMMSCs cultured on SF@MT nanofibers, thereby facilitating improved cell migration and vascular tube formation in HUVECs. The findings from molecular experiments demonstrated that SF@MT nanofibers exerted a stimulating effect on BMMSC-mediated angiogenesis in endothelial cells through the activation of the PI3K/Akt signaling pathway. Furthermore, the *in situ* implantation of SF@MT nanofibers in critical-sized calvarial defects in rats effectively enhanced bone matrix synthesis and facilitated the formation of new blood vessels, thereby expediting the process of vascularized bone regeneration. In summary, the utilization of melatonin-encapsulated SF electrospun nanofibers shows great potential as a viable option for the development of artificial periosteum aimed at repairing critical-sized bone defects.

## CRediT authorship contribution statement

**Lei Deng:** Writing – original draft, Formal analysis, Data curation, Conceptualization. **Mingzhuang Hou:** Writing – original draft, Formal analysis, Data curation, Conceptualization. **Nanning Lv:** Writing – original draft, Formal analysis, Data curation. **Quan Zhou:** Writing – review & editing, Formal analysis, Data curation. **Xi Hua:** Writing – review & editing, Data curation. **Xiayu Hu:** Writing – review & editing, Data curation. **Xiaoyang Ge:** Writing – review & editing, Data curation. **Xuesong Zhu:** Writing – review & editing, Methodology. **Yong Xu:** Writing – review & editing, Methodology. **Huilin Yang:** Writing – review & editing, Methodology, Funding acquisition. **Xi Chen:** Writing – review & editing, Supervision, Investigation, Funding acquisition. **Hao Liu:** Writing – review & editing, Writing – original draft, Supervision, Methodology, Investigation, Conceptualization. **Fan He:** Writing – review & editing, Writing – original draft, Supervision, Resources, Project administration, Methodology, Investigation, Formal analysis, Conceptualization.

## Declaration of competing interest

The authors declare that they have no known competing financial interests or personal relationships that could have appeared to influence the work reported in this paper.

## Data Availability

Data will be made available on request.
