# Supplementary material for: Melatonin-encapsuled silk fibroin electrospun nanofibers promote vascularized bone regeneration through regulation of osteogenesis-angiogenesis coupling
Source: Mater Today Bio. 2024 Feb 2;25:100985. doi: 10.1016/j.mtbio.2024.100985 (PMC10850961; doi:10.1016/j.mtbio.2024.100985)
Supplement: Multimedia component 1 [file mmc1.docx]

**Supplementary Experimental Procedures**

***Cell viability assay***

BMMSCs were seeded into a 24-well plate at a density of 1×10^4^ cells/cm^2^ and exposed to either the nanofiber leachate or PBS (served as a control). Cell viability was assessed using a live/dead cell assay kit (Beyotime). On days 1, 3, and 5, the cells were incubated with a staining reagent at 37°C for 30 min in the absence of light. Following PBS washing, the stained cells were imaged using a fluorescent microscope (Zeiss).

***Cell proliferation***

BMMSCs were seeded into a 96-well plate at a density of 1×10^3^ cells per well and treated with either the nanofiber leachate or PBS. Cell proliferation was evaluated after 1, 3, 5, and 7 days of cultivation using a cell counting kit-8 assay (CCK-8, Beyotime). The CCK-8 solution was added to each well and incubated for 1 h at 37 ℃ in the dark, and the optical density value was measured using a microplate spectrophotometer (BioTek, Winooski, VT, USA) at the wavelength of 450 nm.

***Immunofluorescence staining***

BMMSCs cultured on SF or SF@MT nanofibers were immobilized with a 4% paraformaldehyde solution and permeabilized with 0.1% Triton X-100 (Sigma-Aldrich) for 10 min. Subsequently, the cells were subjected to a blocking step using a 1% bovine serum albumin (BSA) solution for 30 min, followed by incubation with anti-COL1A1 and anti VEGFA antibodies for 1 h. Following a PBS wash, the cells were exposed to a Alexa Fluor® 647 conjugated secondary antibody mixed with phalloidin (Beyotime) for 30 min. The cell nucleus was counterstained using 4΄,6-diamidino-2-phe-nylindole (DAPI, Thermo Fisher Scientific), and digital images were acquired utilizing a fluorescence microscope (Zeiss).

***In Vitro Cell Migration assay***

A density of 2 × 10^5^/well HUVECs was seeded into the upper chamber of a transwell plate (Sigma-Aldrich). Following a 12-h incubation period, non-adherent cells were removed and the remaining cells were fixed using 4% paraformaldehyde for 30 min. The cells were then stained with a 0.1% crystal violet solution (Sigma-Aldrich) for 30 min and observed under an Olympus IX51 microscope. To determine the cell number, a total of 10 randomly selected fields of view were analyzed using the Image J software.

***Scratch wound assay***

HUVECs were seeded in a 6-well plate at a density of 1 ×10^6^/well. Once the cell layer reached 95% confluence, a sterile pipette tip was utilized to create a straight wound. After washing with PBS, the cells were exposed to either the leachate or conditioned medium for 6 or 12 h. At various time points, digital images were captured using an Olympus IX51 microscope and the distance between two edges in the wound area was measured using Image J software.

**Supplementary Figures**

*
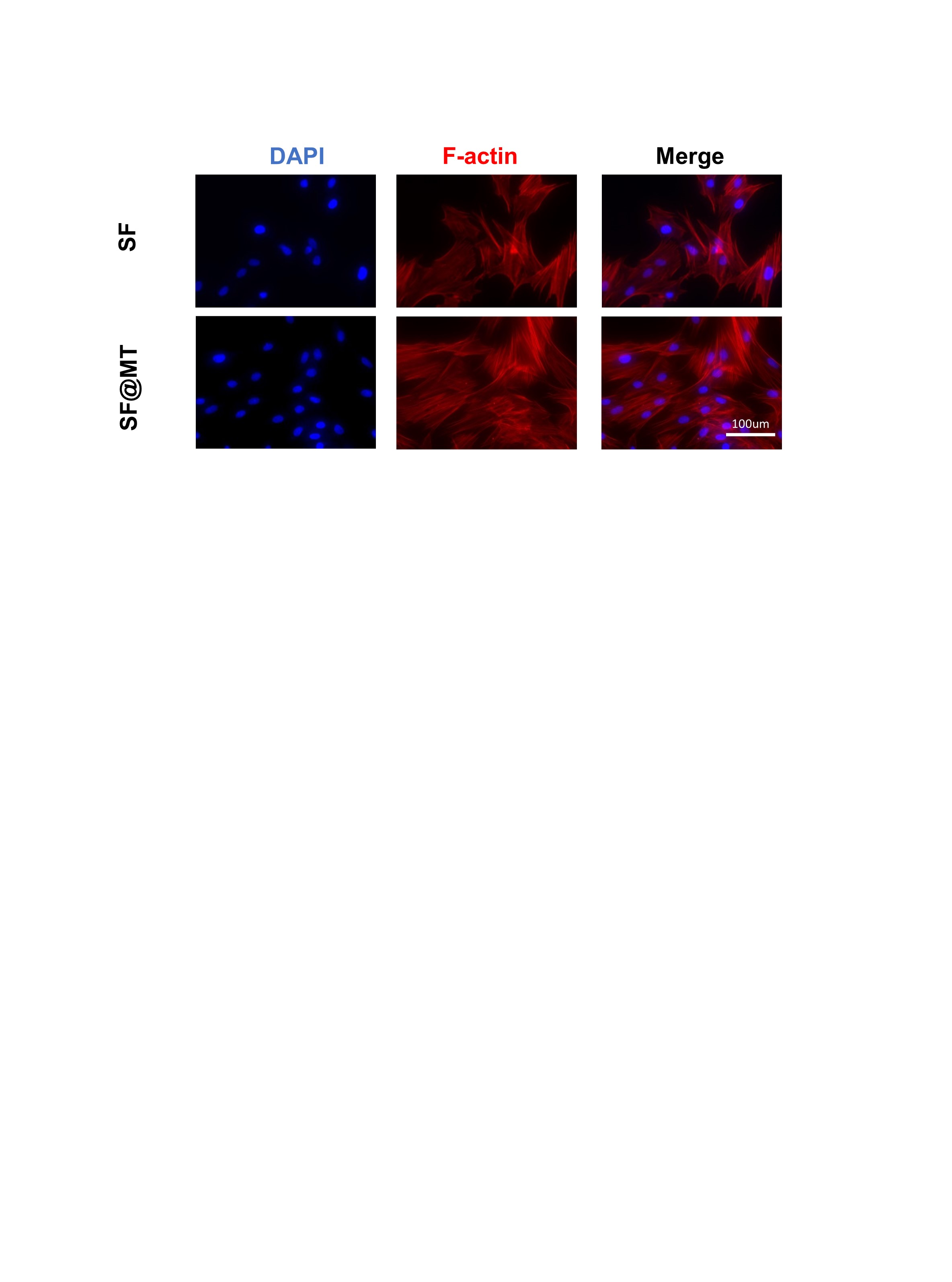
*

**Supple. Fig. 1. Cytoskeleton staining of BMMSCs cultured on SF and SF@MT nanofibers.** BMMSCs cultured on SF and SF@MT electrospun nanofibers exhibited comparable and well-spread cell morphology.

*
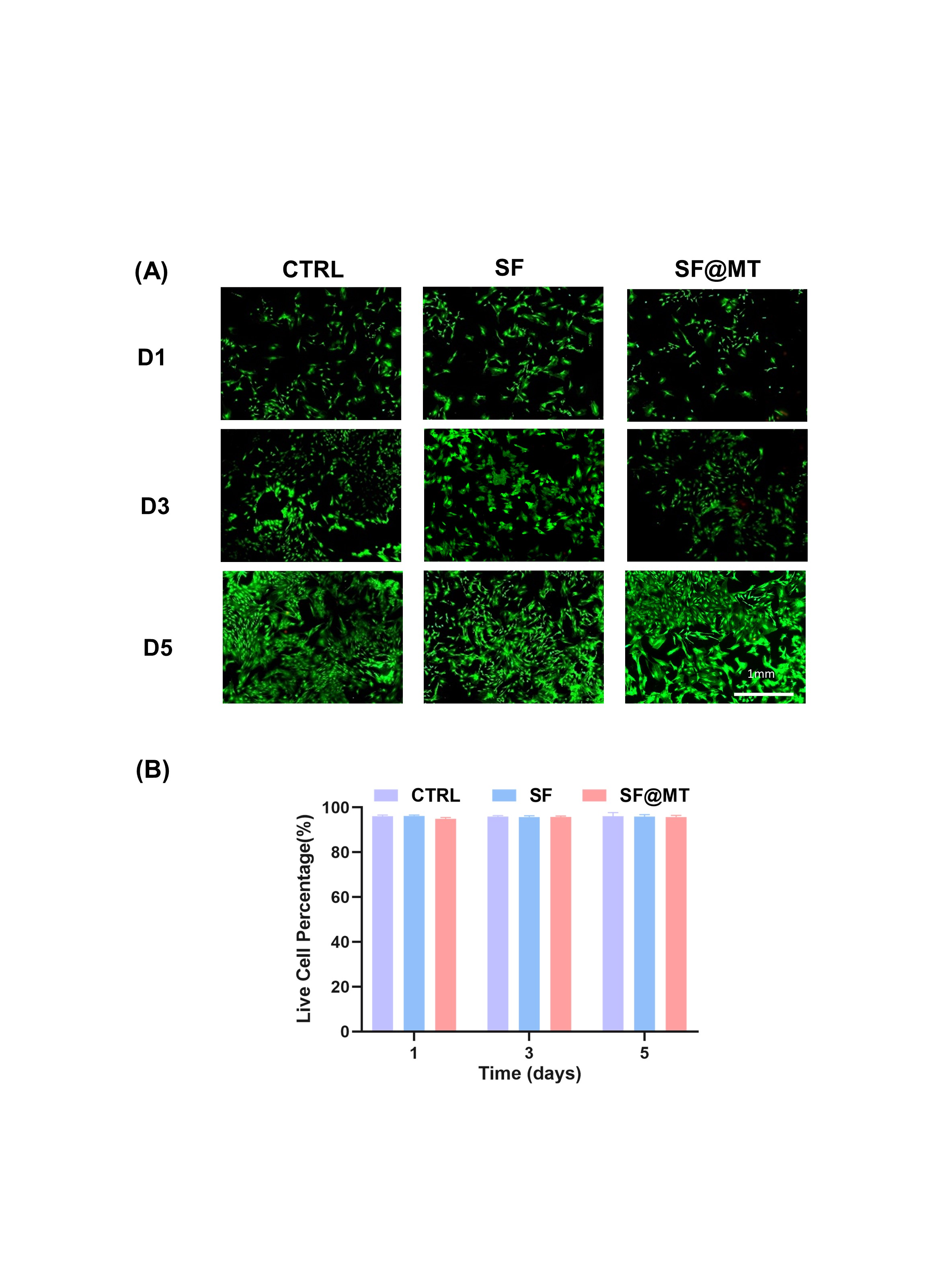
*

**Supple. Fig. 2. Biocompatibility assessment of SF and SF@MT electrospun nanofibers.** (A) Live/Dead staining of BMMSCs treated with the leachate from SF and SF@MT nanofibers. (B) Quantitation of the percentage of live cells, n = 3. The proportions of viable cells in all three groups were similar, indicating the absence of cytotoxicity towards BMMSCs. Data are presented as means ± SD.


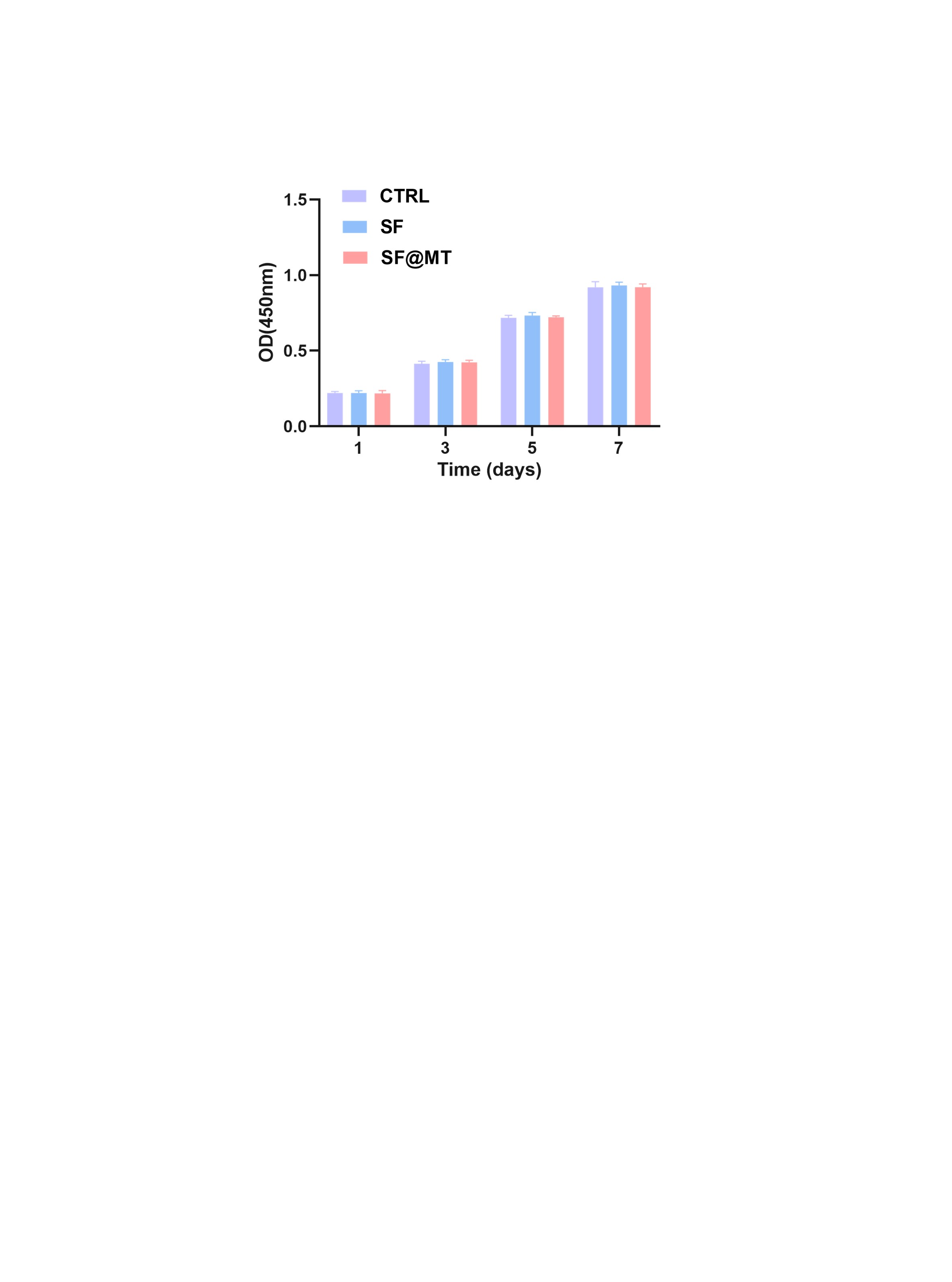


**Supple. Fig. 3. The effect of SF and SF@MT electrospun nanofibers on cell proliferation.** CCK-8 assay for cell proliferation on days 1, 3, 5 and 7, n = 6. The results indicated that there was no significant difference in cell proliferation among the three groups, suggesting that both SF and SF@MT nanofibers have favorable biocompatibility. Data are presented as means ± SD.


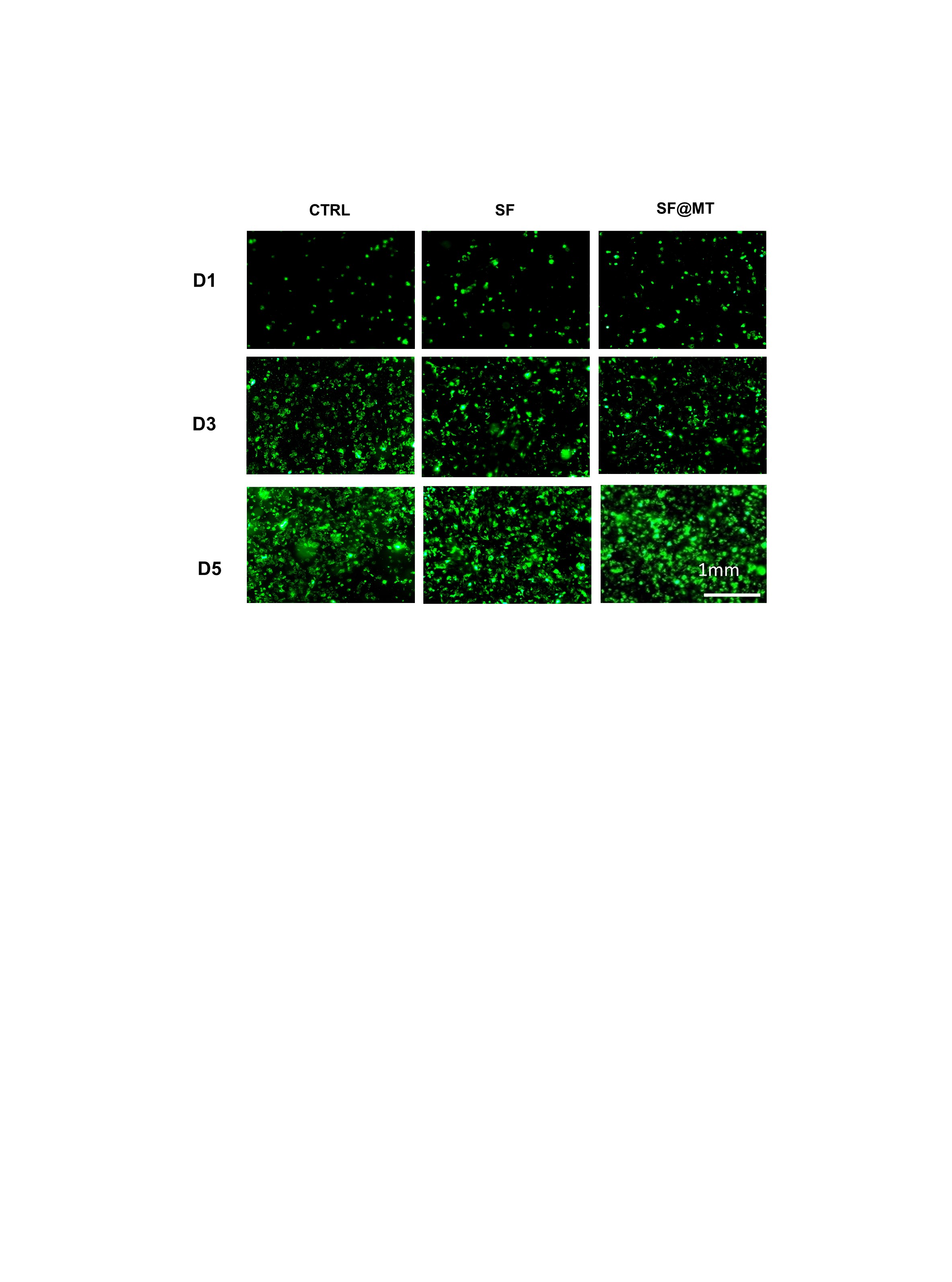


**Supple. Fig. 4. Biocompatibility assessment of SF and SF@MT electrospun nanofibers.** Dio-labeled cells were placed on the nanofibrous membrane, and they demonstrated strong abilities to proliferate and migrate.


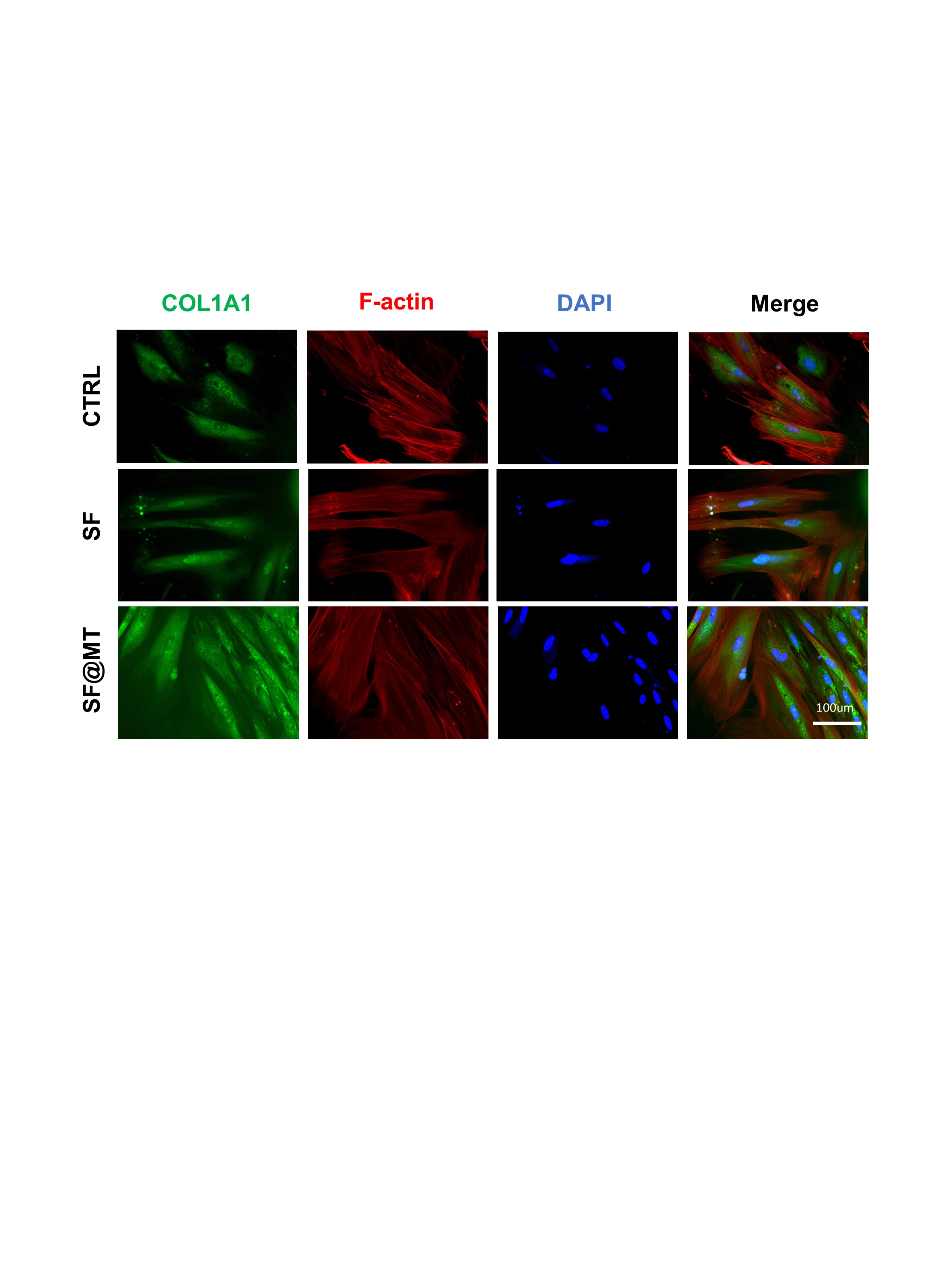


**Supple. Fig. 5.** Representative images of immunofluorescence staining for COL1A1 expression**.** Strongly positive staining for COL1A1 in the SF@MT group suggested that SF@MT promoted the synthesis of osteoblast-specific extracellular matrix.


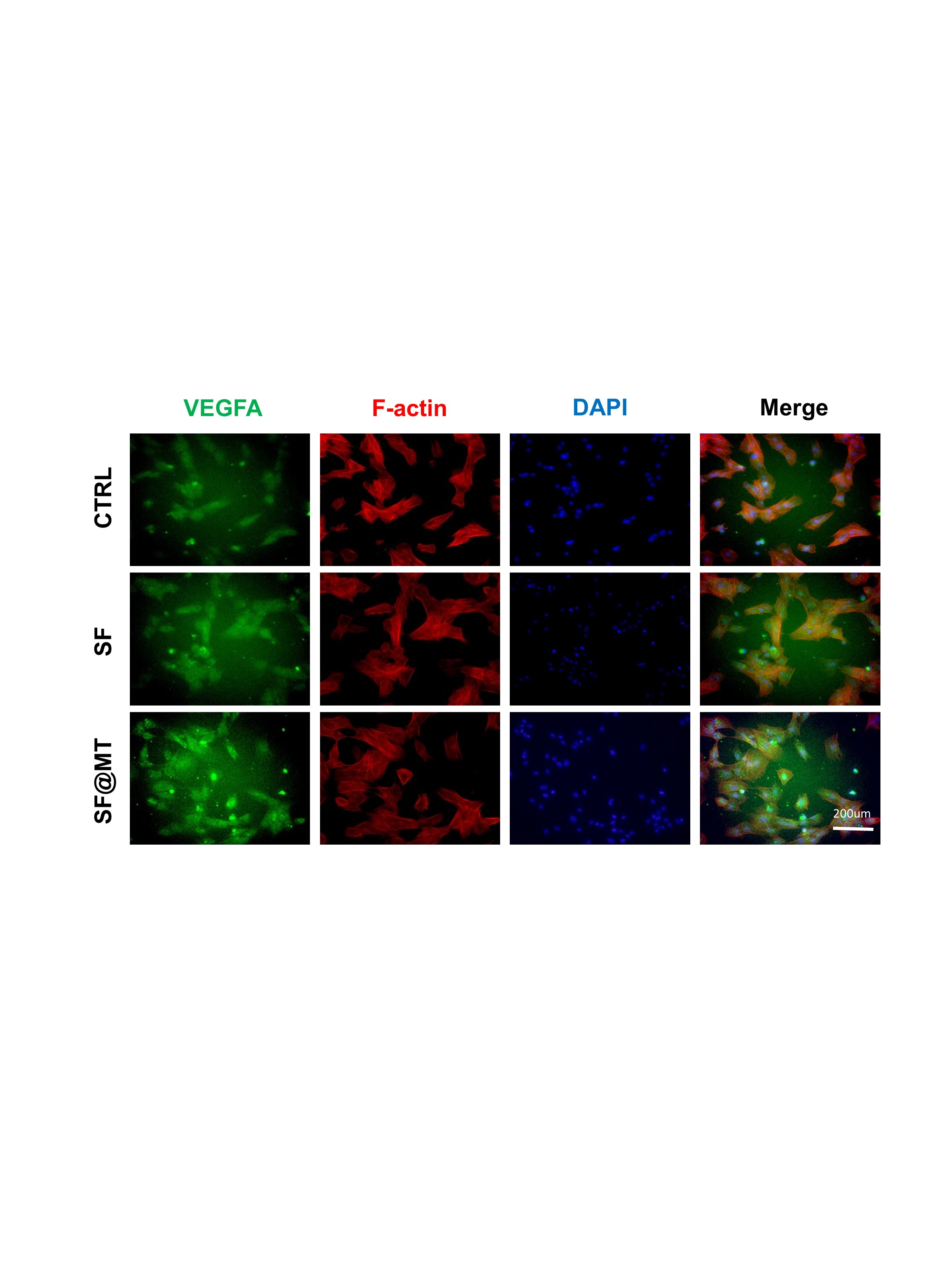


**Supple. Fig. 6.** Representative images of immunofluorescence staining for VEGFA expression**.** BMMSCs cultured on SF@MT nanofibers showed a high level of VEGFA expression, consistent with its mRNA expression.


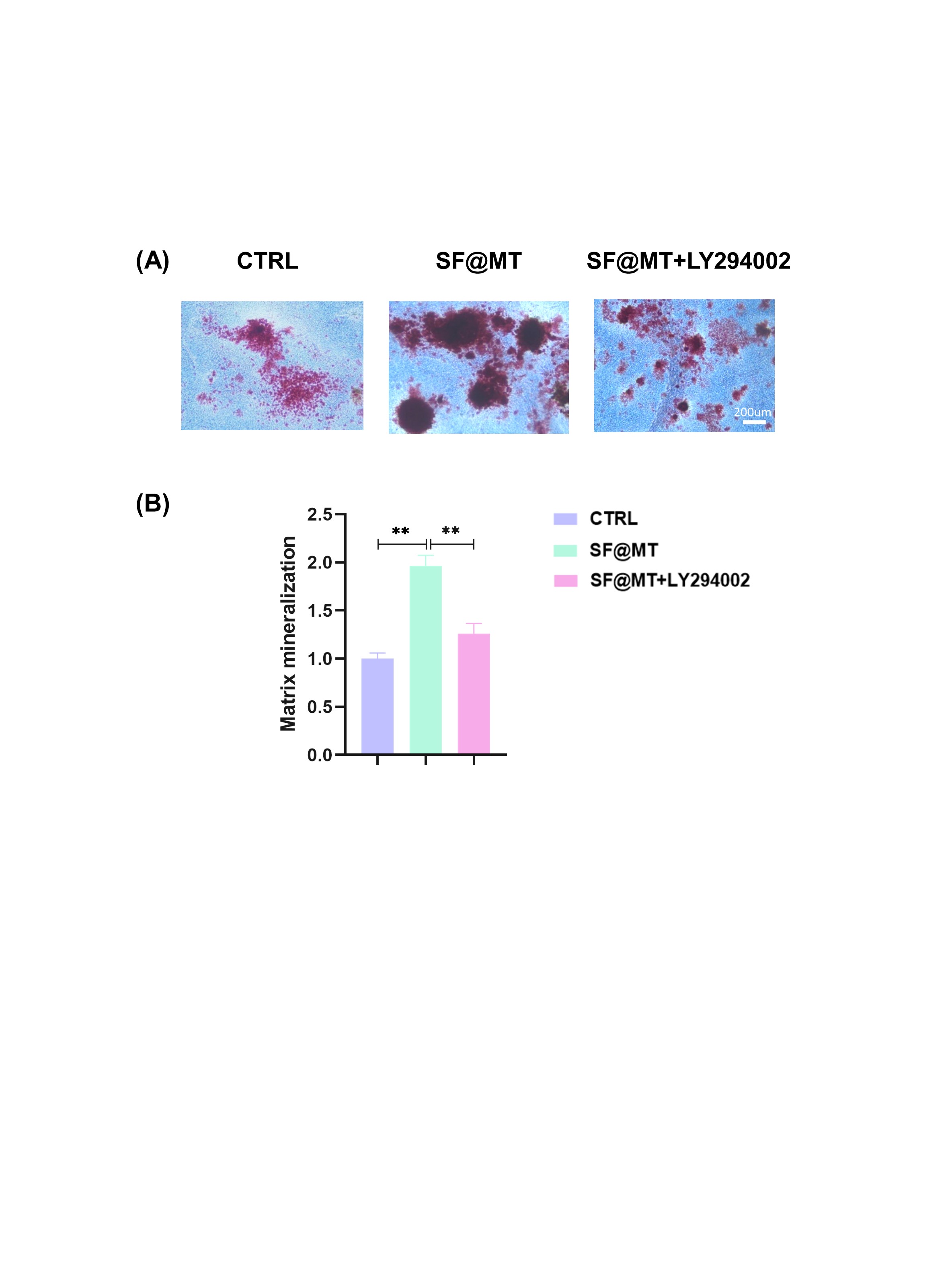


**Supple. Fig. 7.** Inhibition of PI3K/AKT by LY294002 abolished the effect of SF@MT nanofibers on the osteogenesis of BMMSCs. (A) Alizarin Red S (ARS) staining was performed after 21 days of induction. (B) Quantification of the stained mineral layers indicated that LY294002 treatment suppressed matrix mineralization, n=3. Data are presented as means ± SD. Statistically significant differences are indicated by * where *P* < 0.05 or ** where *P* < 0.01 between the indicated groups.


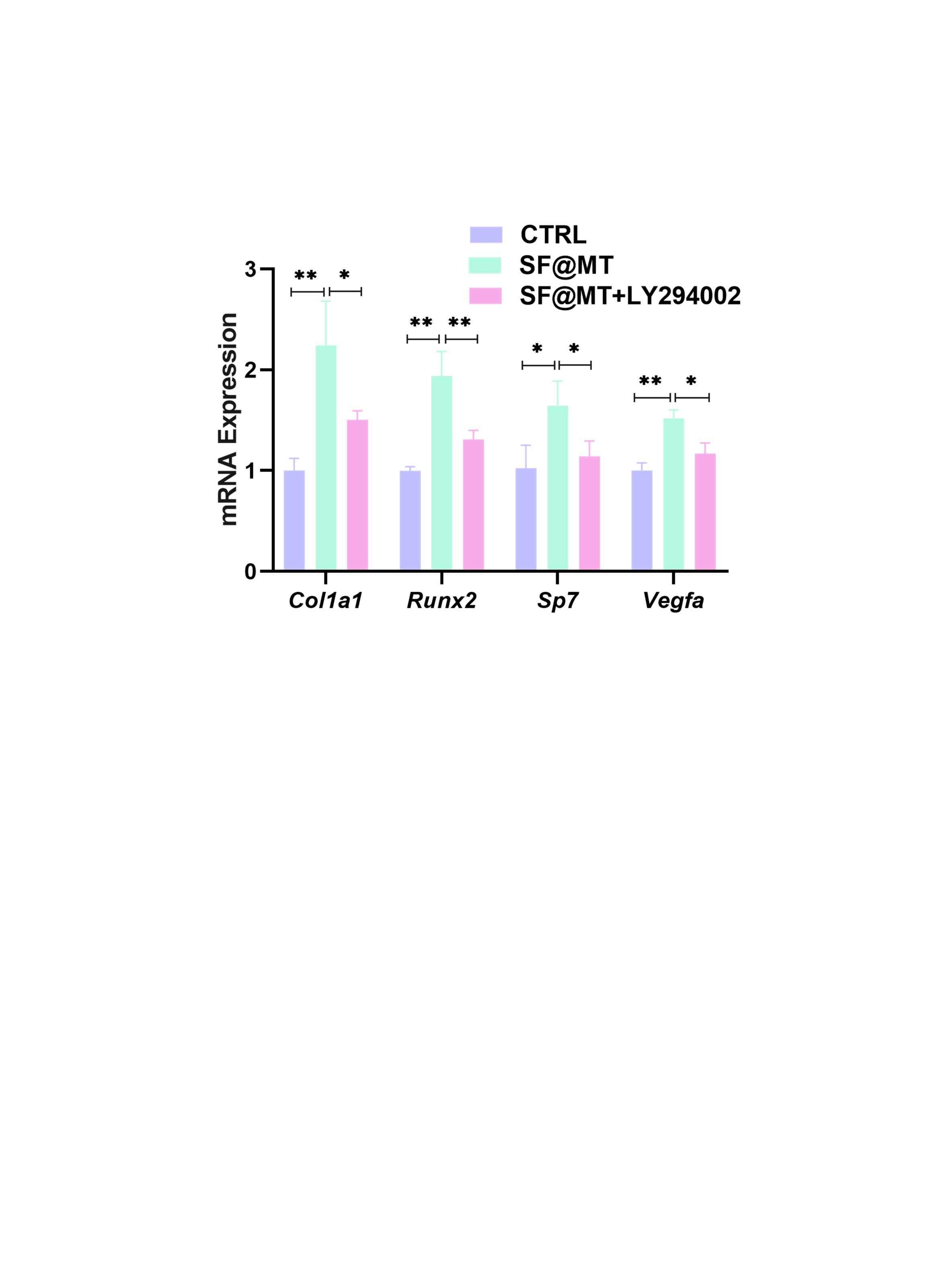


**Supple. Fig. 8.** Inhibition of PI3K/AKT by LY294002 abolished the effect of SF@MT nanofibers on the osteogenesis of BMMSCs. The gene expression of osteogenic makers, including *Col1a1*, *Runx2*, *Osterix*, and *Vegfa*, was determined by RT-PCR, n=4. Data are presented as means ± SD. Statistically significant differences are indicated by * where *P* < 0.05 or ** where *P* < 0.01 between the indicated groups.

**Supplementary Table 1.** Primers used for quantitative real-time RT-PCR.

| Gene | Forward primer sequence (5' -3') | Reverse primer sequence (5'-3') |
| --- | --- | --- |
| *Gapdh* | CAAGTTCAACGGCACAG | CGCCAGTAGACTCCACGAC |
| *Col1a1* | TGTTGGTCCTGCTGGCAAGAATG | GTCACCTTGTTCGCCTGTCTCAC |
| *Runx2* | CCAACTTCCTGTGCTCCGTG | GTGAAACTCTTGCCTCGTCCG |
| *Sp7* | CCCAACTGTCAGGAGCTAGAG | GATGTGGCGGCTGTGAAT |
| *Vegfa* | CACGACAGAAGGGGAGCAGAAAG | GGCACACAGGACGGCTTGAAG |

**Supplementary Table 2.** Antibodies used for Western blot.

| Antibody | Vendor | Catalog Number | Dilution |
| --- | --- | --- | --- |
| COL1A1 | Abcam | ab260043 | 1:2000 |
| RUNX2 | Abcam | ab92336 | 1:2000 |
| SP7 | Abcam | ab209484 | 1:2000 |
| VEGFA | Abclonal | A12303 | 1:2000 |
| p-PI3K | Immunoway Biotechnology | ab219822 | 1:2000 |
| PI3K | Immunoway Biotechnology | ab137040 | 1:2000 |
| p-AKT | Abcam | Ab8805 | 1:2000 |
| AKT | Abcam | ab179463 | 1:2000 |
| β-actin | Abcam | ab8227 | 1:2000 |
| goat anti-rabbit IgG (H&L) | Abcam | ab150079 | 1:10000 |
